# Red-shifted d-luciferin analogues and their bioluminescence characteristics

**DOI:** 10.1039/d5cb00287g

**Published:** 2025-11-14

**Authors:** Pratchaya Watthaisong, Chadaporn Kantiwiriyawanitch, Watcharapa Jitkaroon, Aisaraphon Phintha, Ittiphat Klayparn, Narin Lawan, Philaiwarong Kamutira, Daisuke Sasaki, Surawit Visitsatthawong, Somchart Maenpuen, Ruchanok Tinikul, Jeerus Sucharitakul, Ryo Nishihara, Kazuki Niwa, Yoshihiro Nakajima, Yoshihiro Ohmiya, Pimchai Chaiyen

**Affiliations:** a Biomolecular Science and Engineering (BSE), Vidyasirimedhi Institute of Science and Technology (VISTEC) Wangchan Valley Rayong 21210 Thailand pimchai.chaiyen@vistec.ac.th; b Department of Chemistry, Faculty of Science, Chiang Mai University Chiang Mai Thailand; c Health and Medical Research Institute, National Institute of Advanced Industrial Science and Technology (AIST) Takamatsu Kagawa 761-9035 Japan; d Department of Biochemistry, Faculty of Science, Burapha University, Bangsaen Chon Buri District Chon Buri 20131 Thailand; e Department of Biochemistry, Faculty of Science, Mahidol University Ratchathewi Bangkok 10400 Thailand; f Department of Biochemistry, Faculty of Science, Chulalongkorn University Pathum Wan Bangkok 10330 Thailand; g Health and Medical Research Institute, National Institute of Advanced Industrial Science and Technology (AIST) Tsukuba Ibaraki 305-8566 Japan; h National Metrology Institute of Japan, National Institute of Advanced Industrial Science and Technology (AIST) Tsukuba Ibaraki 305-8563 Japan; i Department of Biomedical Engineering, Osaka Institute of Technology (OIT) Osaka Japan

## Abstract

d-Luciferin (d-LH_2_) is the most used substrate for beetle luciferases in various bioluminescence applications. Here, we successfully synthesized six d-LH_2_ analogues including 5′,7′-dimethoxy-d-LH_2_ and 7′-methylnaphthol-d-LH_2_ as novel compounds. We also developed a continuous one-pot green synthesis method to improve yields of luciferins from condensation of quinone and d-Cys (63-fold greater than the previous report). The novel d-LH_2_ analogues were tested with five luciferases (Fluc, SLR, Eluc, Pmluc-WT, and Pmluc-N230S), and all the compounds emitted bioluminescence at wavelengths longer than that of d-LH_2_ (>80 nm). The reaction of SLR with 5′,7′-dimethoxy-d-LH_2_ gave the longest red-shifted bioluminescence at 663 nm. Remarkably, the reactions of 5′-methyl-d-LH_2_ emit longer wavelengths and brighter light than those of d-LH_2_ in all tested luciferases, except for Eluc. Interestingly, the novel red-shifted 5′,7′-dimethyl-d-LH_2_ also provided prolonged bioluminescence with a rate of light decay slower than that of d-LH_2_. We further demonstrated applications of 5′-methyl-d-LH_2_ and 5′,7′-dimethyl-d-LH_2_ in mammalian cell lines expressing Fluc, SLR, and Pmluc-N230S. 5′-Methyl-d-LH_2_ provided about 11.2-fold greater sensitivity to detect Fluc in the HEK293T crude lysate than d-LH_2_, achieving the detection with a lower number of cell lines. The red-shifted 5′,7′-dimethyl-d-LH_2_ also exhibits high sensitivity when using a red light filter to monitor live cell bioluminescence. These d-LH_2_ analogues, 5′-methyl-d-LH_2_ and 5′,7′-dimethyl-d-LH_2_, are promising substrates for future cell-based assays and real-time monitoring applications.

## Abstract 2

The red-shifted 5′-Me-d-LH_2_ and 5′,7′-Me-d-LH_2_, are promising substrates for future cell-based assays and *in vivo* imaging applications.

## Graphical abstract

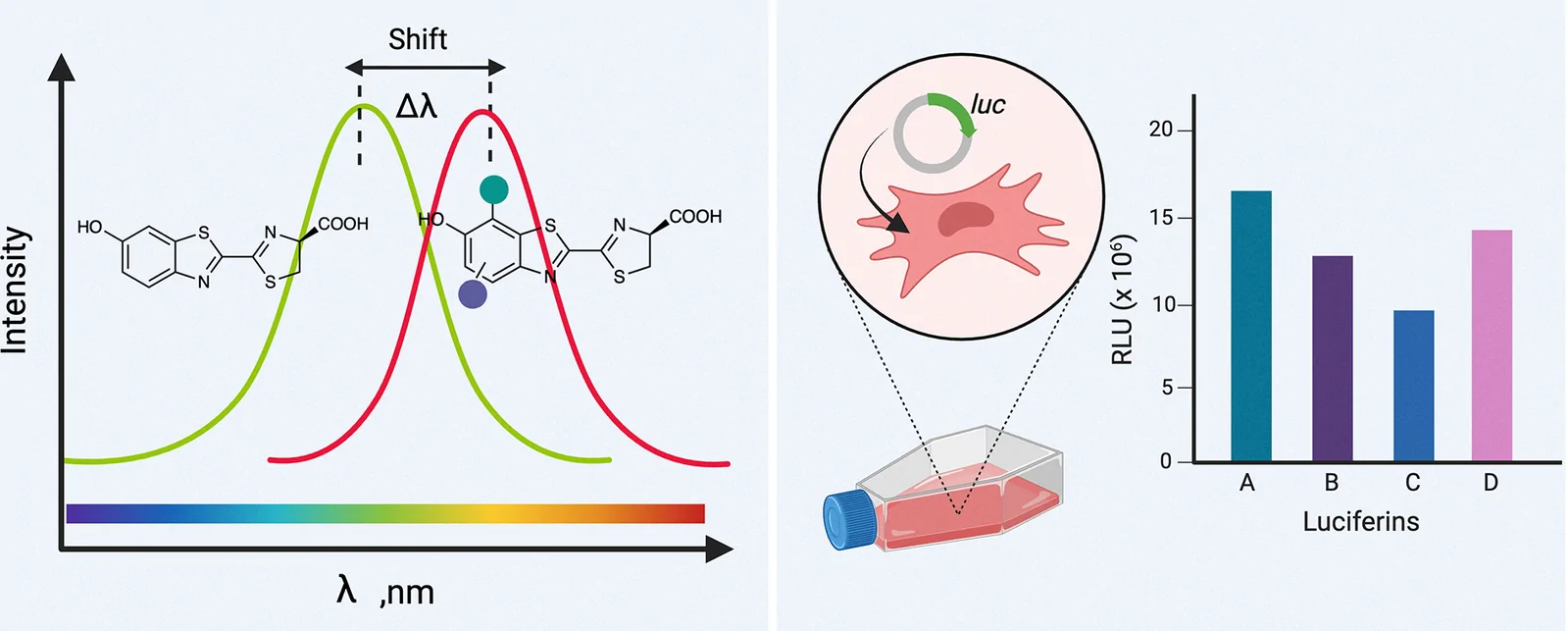

## Introduction

Reactions of luciferases and luciferins found in various organisms can generate cold light emission or bioluminescence (BL). These reactions are efficient analytical and visualization tools which have been applied in various detection technologies.^1^ It has been estimated that nearly 30% of high-throughput screening (HTP) reactions used in academia and industry involve luciferase reactions.^2^ Beetle luciferases, especially firefly luciferases (Fluc), are among the most widely used BL systems in biochemical and cell-based assays because of their catalytic oxidation of the d-luciferin (d-LH_2_) substrate to oxyluciferin generates green-yellow light (∼560 nm) with a high quantum yield (41%).^2,3^ Although the Fluc reaction is useful for a variety of assays, its application for *in vivo* imaging is limited because BL signals generated by the natural d-LH_2_ emits green-yellow light which does not penetrate well into animal cells or tissues. The current use of d-LH_2_ in animal experiments requires the usage of high doses, thus resulting in high experimental cost.^4,5^d-LH_2_ analogues with red-shift and steady BL are thus valuable for improving Fluc applications because this would allow better light penetration through the cells, blood and tissues of animal models.^6^

Numerous d-LH_2_ analogues particularly in the forms of 6′-aminoluciferin (6′-NH_2_LH_2_) and cyclic alkylaminoluciferin (CycLuc1) emit red-shifted BL.^7^ The longest wavelengths could be achieved with AkaLumine–HCl and its derivatives including seMpai (up to 675 nm)^8^ and infraluciferin (iLH_2_, >700 nm).^7,8^ However, their applications are still challenging because the natural Fluc (the most widely used luciferase for general applications) does not utilize these d-LH_2_ analogues well. Several enzyme engineering campaigns were carried out to evolve luciferases suitable for using these compounds and increase the BL intensity.^8–10^ Although the engineered luciferase, namely Akaluc, could use AkaLumine–HCl as a substrate and exhibit brighter BL than the native Fluc,^7,11^ the overall signals are still low.

This is due to the low quantum yield of AkaLumine–HCl with Fluc (4.0 ± 0.5%), which is significantly lower than that of natural d-LH_2_.^12^ Therefore, new red-shifted d-LH_2_ analogues which can be used as dropped-in substrates for the native beetle luciferases would provide alternative choices for biomedical researchers to directly use the widely available beetle luciferase systems to generate red-shifted BL for cell-based screening applications.

Currently, the synthesis of d-LH_2_ and most of its analogues at a scale of >50 mg can only be performed through chemical methods which typically require multi-step organic synthesis and purification (∼4 steps). The chemical reactions need to be carried out at 120–190 °C using hazardous chemicals such as acetic anhydride (Ac_2_O), pyridine, sulfolane, and dimethylformamide (DMF)^13–15^ by highly skilled chemist experts. We previously reported the chemo-enzymatic synthesis of novel d-LH_2_ analogues with methyl substituent groups at the benzothiazole ring of d-LH_2_ which gave a red shift in emission up to 620 nm with good BL intensity and light stability. These compounds can be used directly as substrates for the native Fluc and have been demonstrated as environmental monitoring tools without Fluc engineering.^16^ Our methodology was based on the condensation of benzoquinone (BQ) and d-cysteine (d-Cys) to form the d-LH_2_ using the HadA enzyme (HELP, HadA Enzyme for Luciferin Preparation);^17,18^ the process was optimized to obtain a high yield of d-LH_2_ analogues of about ∼51% with a 7.1 mg yield at the 0.5 L scale.^16^ Although the HELP process is green, its scale up is still limited when compared to other conventional chemical synthesis methods because it utilizes the HadA and associated enzymes.

**Fig. 1:**
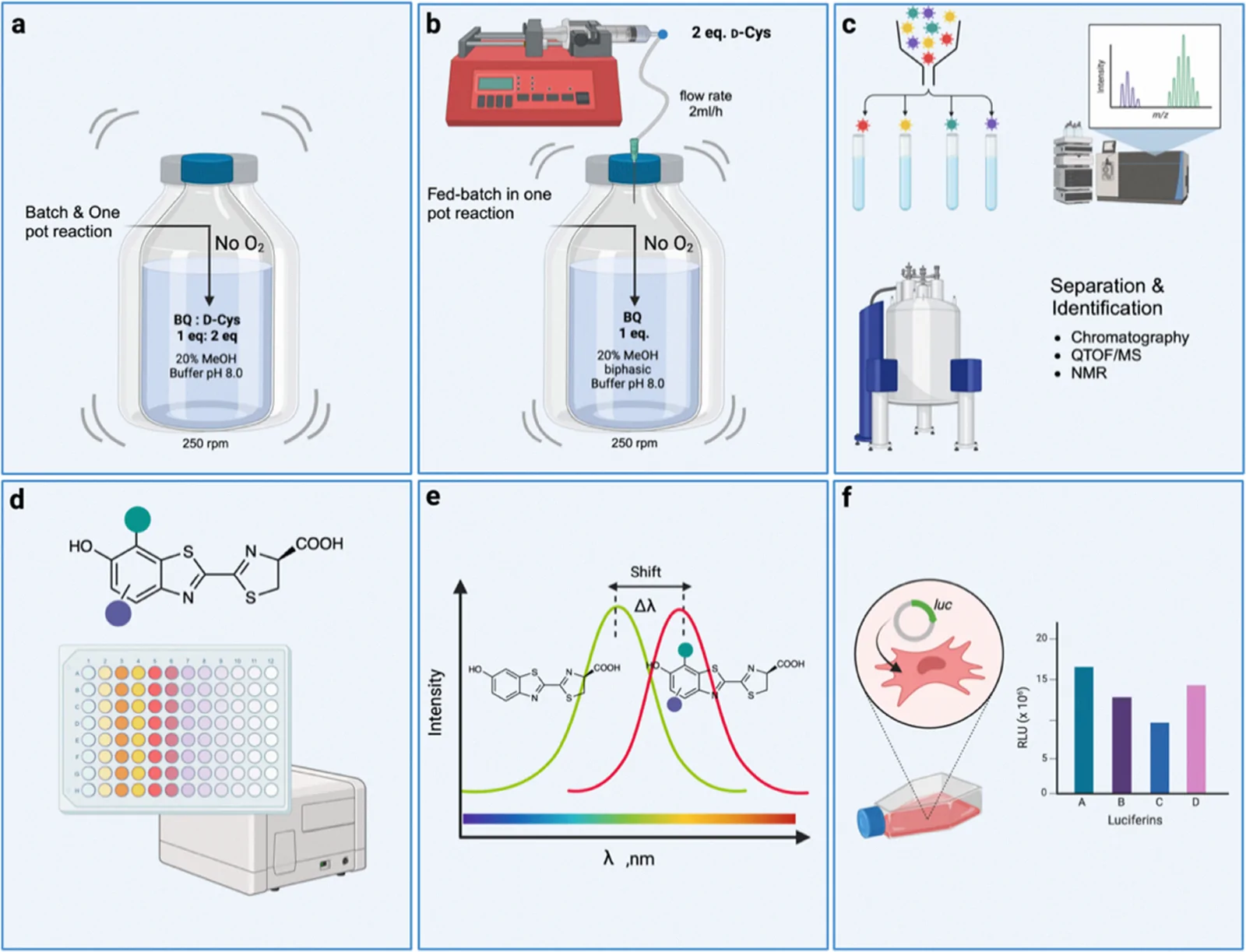
Graphical representation of the overall workflow. (a) Batch and one-pot reaction for d-LH_2_ synthesis. (b) Fed-batch synthesis of d-LH_2_ analogues in one-pot reaction. (c) d-LH_2_ analogues were isolated and characterized and their structures were elucidated by ^1^H and ^13^C NMR and high-resolution mass spectrometry. (d) BL reactions of new d-LH_2_ analogues with five types of beetle luciferases. (e) BL characteristics of novel d-LH_2_ analogues. (f) Demonstration of applications for new d-LH_2_ analogues in mammalian cell line assays and real-time bioluminescence monitoring of live cells.

In this work, we developed a cost-effective one-pot green synthesis procedure for methyl-, methoxy-, and naphthol-d-LH_2_ analogues using the condensation reaction of BQ derivatives and d-Cys without adding the HadA and auxiliary enzymes. The process was optimized to improve the product yield for the bulk scale and easy preparation of d-LH_2_ analogues while providing compatibility with green chemistry principles^19^ (Fig. 1a). We have used the method to prepare six methyl-, methoxy and naphthol-substituted d-LH_2_ analogues including natural d-LH_2_ (2a), 5′-methyl-d-LH_2_ (5′-MeLH_2_, 2b),^16^ 5′,7′-dimethyl-d-LH_2_ (5′,7′-DiMeLH_2_, 2c),^20^ and 4′,7′-dimethyl-d-LH_2_ (4′,7′-DiMeLH_2_, 2e)^21^ with two novel luciferins: 5′,7′-dimethoxy-d-luciferin (5′,7′-DiOMeLH_2_, 2d) and 7′-methylnaphthol-d-luciferin (7′-MeNpLH_2_, 2f). With an appropriate substrate ratio and continuous addition of substrates under anaerobic conditions, the developed one-pot green synthesis approach can preserve the stability of BQ derivatives and d-Cys, preventing substrate deterioration, reducing byproduct formation and increasing d-luciferin analogue yield (by 63-fold relative to that of the previously reported chemical condensation method,^17^Fig. 1b and 1c). The synthesized d-LH_2_ analogues (2a–2f) were tested with five known beetle luciferases including *Photinus pyralis* luciferase (Fluc), *Phrixothrix hirtus* red-emitting luciferase (SLR), *Pyrearinus termitilluminans* emerald luciferase (Eluc), *Pyrocoelia miyako* luciferase wild-type (Pmluc-WT), and the *Pyrocoelia miyako* luciferase variant (Pmluc-N230S) commonly used in BL applications^22–26^ (Fig. 1d). Their biochemical, steady-state kinetics and BL properties of these enzymatic reactions were characterized (Fig. 1e and f). The novel methyl- and methoxy-d-LH_2_ analogues demonstrated their better red-shifted BL (>600 nm), increased brightness, and slow decay rate of BL emission compared to the reaction of natural d-LH_2_ (2a). 5′-Methyl-d-LH_2_ and 5′,7′-dimethyl-d-LH_2_ which showed promising BL characteristics in enzymatic reactions were further explored for their *in vivo* BL properties in mammalian cell lines expressing Fluc, SLR, and Pmluc-N230S. These newly discovered unique properties of methyl-d-LH_2_ compounds provide the unique BL characteristics advantageous for future cell-based assays and real-time monitoring applications.

## Results and discussion

### Synthesis of d-LH_2_ analogues and their yield optimizations

**Fig. 2:**
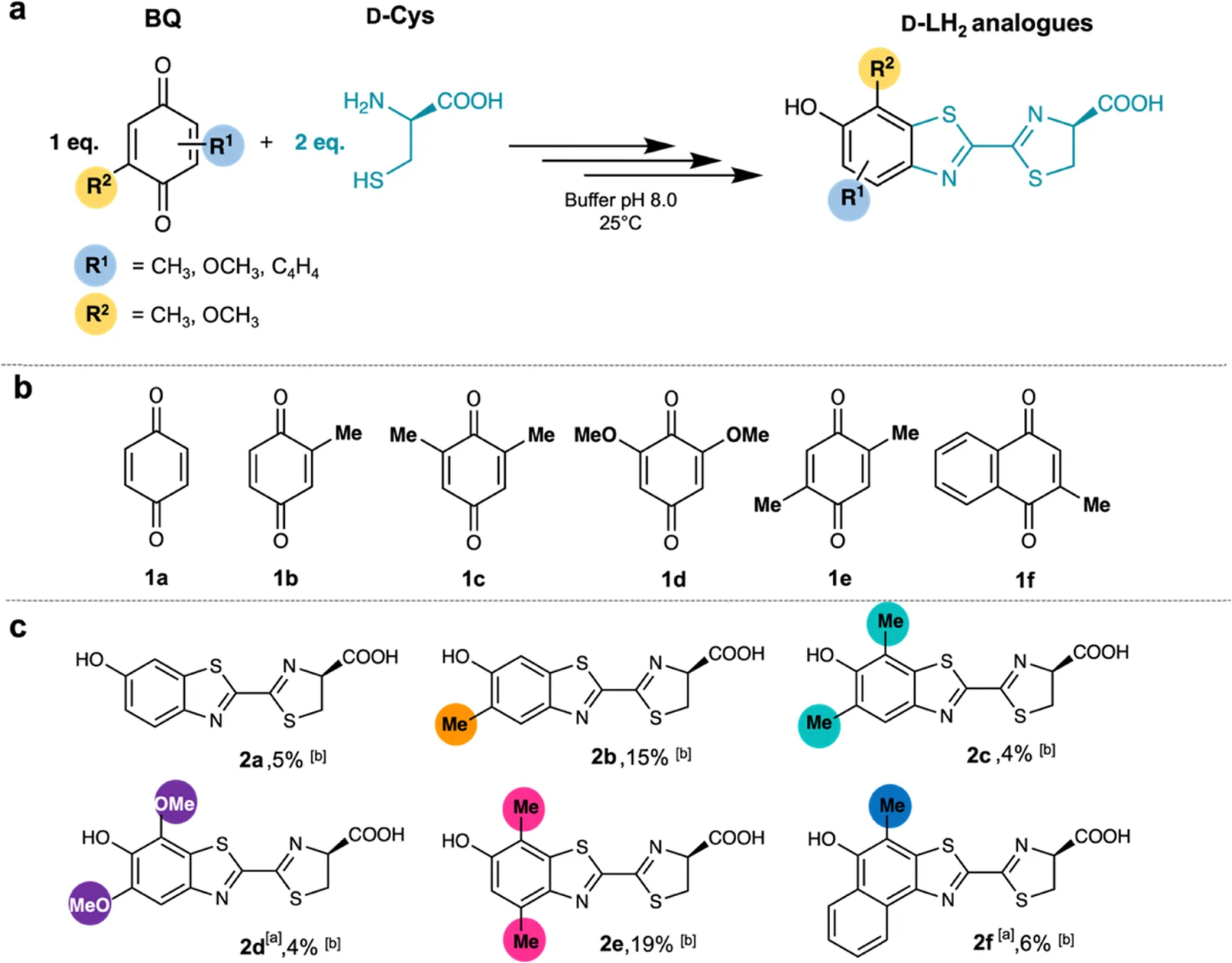
Synthesis of d-LH_2_ and its analogues by condensation of d-Cys and BQ derivatives. (a) d-LH_2_ and its derivatives could be obtained under batch conditions. (b) BQ derivatives (1a–1f) serve as substrates for synthesizing d-LH_2_ analogues. (c) The d-LH_2_ analogues (2a–2f) obtained from condensation of d-Cys and BQ derivatives (1a–1f). [a] Novel d-LH_2_ analogues are 5′,7′-DiOMeLH_2_ (2d) and 7′-MeNpLH_2_ (2f). [b] Isolated yields of d-LH_2_ analogues after purification.

We investigated the condensation of various BQ (1a) derivatives and d-Cys to synthesize d-LH_2_ analogues with more red-shifted light emission (Fig. 2a). High-resolution quadrupole time of flight mass spectrometry (QTOF-MS) was used for identifying potential products. The results (Table S1) revealed that the BQ derivatives (1a–1f) can serve as electrophilic substrates (Fig. 2b) for the d-Cys condensation reaction, producing the d-LH_2_ analogues (2a–2f) shown in Fig. 2c. Structures of the novel d-LH_2_ analogues were elucidated by LC/QTOF-MS and NMR (Tables S1–S3) as 5′,7′-DiOMeLH_2_ (2d) and 7′-MeNpLH_2_ (2f), as shown in Fig. 2c. These compounds were not previously found in nature nor reported.

**Fig. 3:**
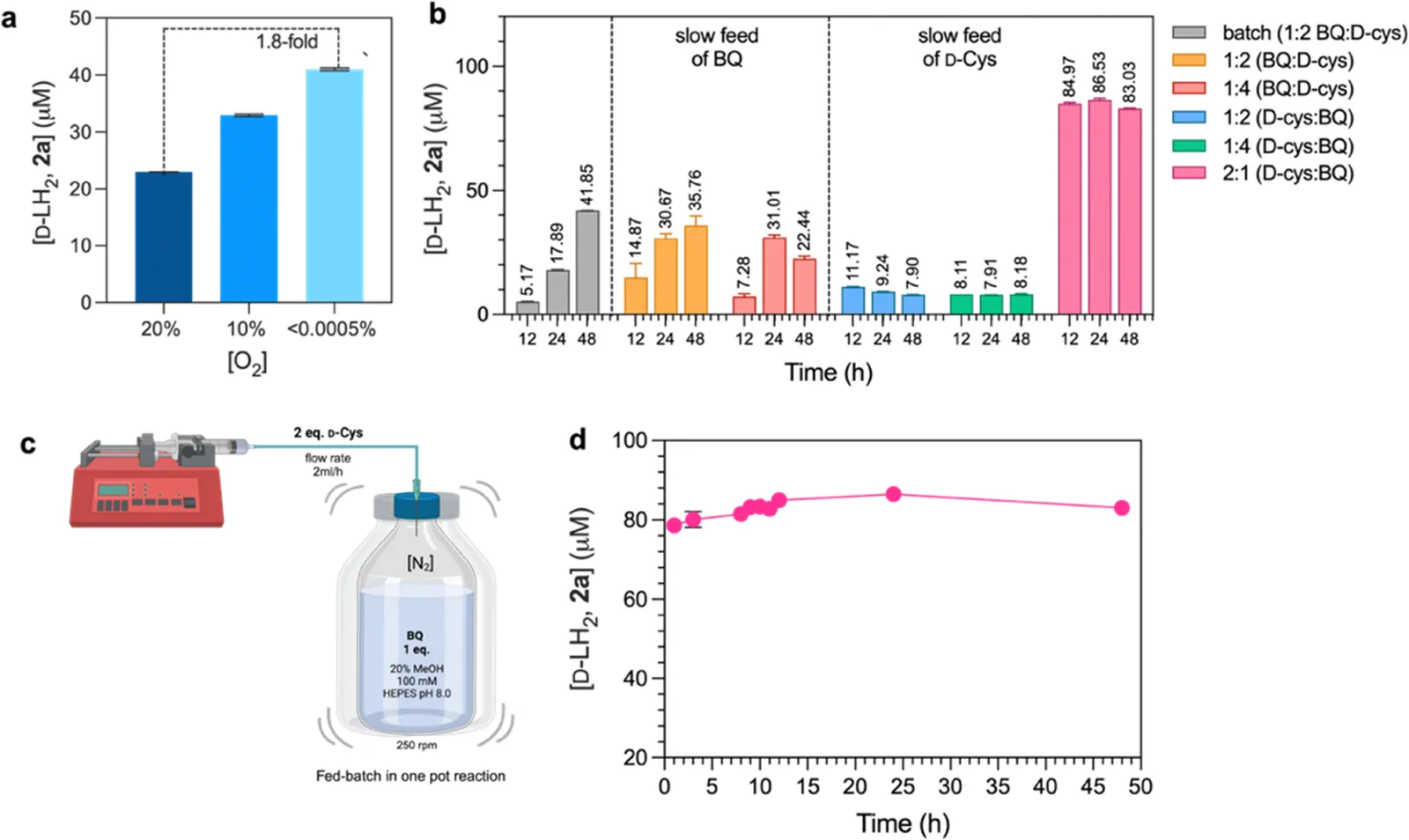
Optimization of 2a production yield. (a) Effects of O_2_ concentrations on 2a formation. (b) Synthesis of 2a by pumping a solution of one substrate into another substrate solution with different ratios under anaerobic conditions. (c) The synthesis of 2a could be set up by continuously feeding 2 eq. of d-Cys (2 mL h^−1^) into 1 eq. of 1a under anaerobic conditions. (d) Kinetics of continuous formation of 2a by feeding 2 eq. of d-Cys (2 mL h^−1^) into 1 eq. of 1a under anaerobic conditions.

Previously, the yield of d-LH_2_ (2a) obtained using BQ (1a) and d-Cys condensation alone was poor (0.3%).^17^ We hypothesized that this was due to the instability of 1a. We thus screened for buffers and reaction conditions which might be suitable for providing better yields. Results in Fig. S1 indicate that the organic buffer, 100 mM HEPES, gave the highest d-LH_2_ yield compared to other systems. We further explored co-solvent systems to stabilize 1a and enhance the yield of d-LH_2_ synthesis of 2a. We found that the addition of 20% (v/v) methanol increased the yield of 2a formation by about 2.8-fold compared to the system without any solvent addition (Fig. S2). We further identified a bottleneck of 1a and d-Cys condensation by varying the percentage of oxygen in the reaction. The reaction buffers were prepared by equilibration with various concentrations of O_2_ (<0.0005%, 10%, and 20%) and used in the reactions to monitor the product 2a formed. The results in Fig. 3a reveal that low concentrations of O_2_ tend to improve the yield of 2a formation, possibly by preventing the decay of 1a to another form. With <0.0005% O_2_ (anaerobic condition), the reaction gave the highest yield of 2a formation which was greater than that of the 20% O_2_ (air-saturation) condition by about 1.8-fold with a d-LH_2_ formation rate of 3.26 µM h^−1^ (Fig. S5 and Table S4).

After finding that the instability of the 1a derivatives and d-Cys substrates is a limiting factor for the generation of d-LH_2_ analogues, we designed a flow chemistry strategy by continuously mixing one substrate with another under various conditions. Fig. 3b illustrates the reactions to generate 2a in batch and continuous feeding (fed-batch) systems. Due to the O_2_ vulnerability of the substrates, only low percentages of 2a formation (up to 18%) could be obtained from the batch reactions within 24 h. For the continuous feeding of 1a into the anaerobic buffer containing 2 (orange) and 4 (old rose) equivalents (eq.) of d-Cys, the yield of 2a formation was about twice that of the batch reactions (Fig. 3b, orange bar at 24 h). Remarkably, when 2 equivalents (eq.) of d-Cys were pumped into 1 eq. of 1a under anaerobic conditions, the highest yield of 2a formation (about 86 µM) could be obtained (Fig. 3b, pink bar at 24 h). This yield was 16.4-fold greater than that of the batch reaction after 12 h (Fig. 3b). Therefore, by pumping 2 eq. of d-Cys into a 1 eq. solution of 1a under anaerobic reactions (Fig. 3c), the stability of the substrates could be greatly enhanced and the reaction could be finished after 8 h (Fig. 3d).

To synthesize d-LH_2_ analogues at a semi-large scale, our method described above and the recently published protocol^27^ of using BQ, L-Cys methyl ester and d-Cys to synthesize d-LH_2_ were tested. We found that 2a and 2c compounds could be prepared with 50 and 66% yield, respectively, when using the BQ derivatives (1a and 1c) and the L-Cys methyl ester and d-Cys condensation protocol. However, this protocol could not be used to prepare other d-LH_2_ analogues (compounds 2b and 2d) because it gave a mixture of products (non-specific reactions in the case of 2b synthesis) and the electron-donating group of 1d affected the cyclization of the benzothiazole ring (in the case of 2d synthesis). These compounds could only be prepared using a fed-batch reaction shown in Fig. 3 and described above. The fed-batch protocol developed in this work is thus useful for synthesizing d-LH_2_ analogues without utilizing harmful catalysts or toxic chemicals. The reaction also requires less usage of organic solvent and thus is compatible with green chemistry principles. These findings set an important foundation for future large-scale production of the compounds.

### NMR, mass spectra, absorption, and fluorescence characteristics of d-LH_2_ analogues

The method described in the previous section was used to synthesize d-LH_2_ analogues (2a–2f) from the BQ derivative substrates (1a–1f, Fig. 1b). The products were isolated (purity up to 97%), and their structures were characterized using QTOF-MS and NMR spectroscopy (Fig. 1c and Fig. S3 and S4 and Tables S3 and S4). The observed *m*/*z* with low mass error values of d-LH_2_ analogues (2a–2f) from the QTOF-MS data (Table S1) confirmed that the BQ derivatives (1a–1f) can react with d-Cys to yield the products in one-pot synthesis. The structures of 2a–2f were then elucidated using ^1^H NMR (Fig. S3 and Table S2) and ^13^C NMR (Fig. S4 and Table S3) to identify the molecular structures of the d-LH_2_ analogues. The NMR results indicate that the benzothiazole rings of the d-LH2 analogues were substituted with methyl and methoxy groups at 4′, 5′, and 7′-positions (Fig. 2c) and demonstrated that we could obtain novel compounds including 5′,7′-DiOMeLH_2_ (2d) and 7′-MeNpLH_2_ (2f). All of the d-LH_2_ analogues (2b–2f) were further investigated for their absorption and fluorescence characteristics in comparison with the native d-LH_2_ (2a) as described below.

Absorption and fluorescence characteristics of d-LH_2_ analogues (2a–2f) in 100 mM HEPES pH 8.0 at 25 °C were recorded. The natural d-LH_2_ (2a) displayed a maximum adsorption peak (*λ*_max_) at 327 nm, while 2b which has a methyl group addition at the 5′-position of the benzothiazole moiety also gave a similar *λ*_max_ at 323 nm (Table S6). However, for 2c and 2e which have two methyl substitutions at the benzothiazole skeleton, their absorption spectra shifted to longer wavelengths of *λ*_max_ at 334 and 330 nm, respectively (Table S5). The addition of dimethoxy (2d) and naphthol groups (2f) resulted in longer wavelength shifts (*λ*_max_ at 412 and 437 nm, respectively) (Table S5). Similarly, the fluorescence properties of natural d-LH_2_ (2a) and methyl-d-LH_2_ analogues (2b, 2c, and 2e) showed similar fluorescence emission spectra with peaks around 523–560 nm. For 2d and 2f, their emission spectra showed a greater bathochromic shift with the peaks around 620 and 580 nm, respectively (Table S5). These absorption and fluorescence characteristics of the d-LH_2_ analogues (2a–2f) provide a means by which BL can be red shifted (shown later). It should be noted that their uniqueness in fluorescence is also valuable for future use in florescence-based sensor applications.^28,29^

### Bioluminescence characteristics of d-LH_2_ analogues

**Fig. 4:**
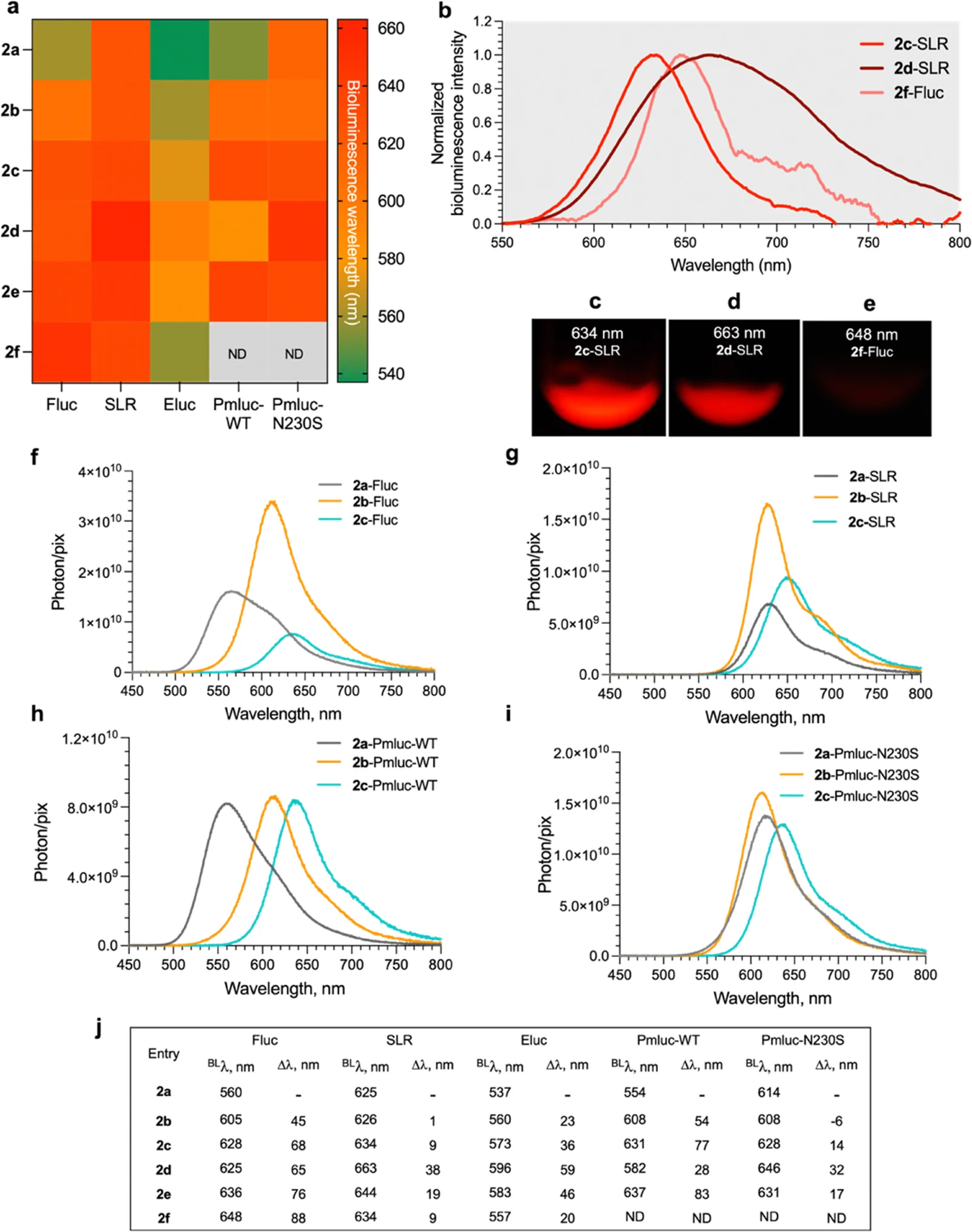
BL characteristics of d-LH_2_ analogues (2a–2f) reacted with various luciferases. Reactions of various luciferases with d-LH_2_ analogues (2b–2f) were carried out at 25 °C in 100 mM HEPES buffer at pH 8.0 and compared to those of natural d-LH_2_ (2a). (a) Heat maps describing BL emission of d-LH_2_ analogues (2a–2f) generated from their reactions with Fluc, SLR, Eluc, Pmluc-WT, and Pmluc-N230S luciferases. (b) BL emission spectra of novel d-LH_2_ analogues (2d and 2f) obtained from their reactions with their orthogonal luciferases. (c–e) BL signals obtained from the reactions of novel d-LH_2_ analogues (2d and 2f) and their orthogonal luciferases were recorded by a DSLR camera. (f–i) The BL emission spectra of the luciferase (5 µM)–luciferin (2 µM) reactions were measured using the integrating sphere-based multichannel spectrometer. (j) The difference in emission wavelengths of d-LH_2_ analogues (2b–2f) compared to those of the natural d-LH_2_. ND denotes the non-detectable signals.

The purified d-LH_2_ analogues (2a–2f) were investigated for their BL characteristics using known luciferases, *i.e.*, Fluc, SLR, Eluc, Pmluc-WT, and Pmluc-N230S. Results in Fig. 4a showed that 2b–2f could serve as substrates for various luciferases (except 2f, which could not serve as a substrate for Pmluc-WT and Pmluc-N230S) and generate light emission with longer wavelengths (red-shifted) compared to the natural d-LH_2_ (2a). Interestingly, the pairing of novel d-LH_2_ analogues (2d and 2f) with SRL and Fluc gave red light emissions at longer than 630 nm which could be recorded by a digital single-lens reflex (DSLR) camera (Fig. 4b–e). We found that a methyl substituent in the benzothiazole ring of d-LH_2_ could also modulate absorption, fluorescence and BL characteristics of luciferases (Table S5). Particularly, the BL emission was altered from yellow-green (∼560 nm) of the natural d-LH_2_ (2a) to be in the red-shifted region (>600 nm).

We also compared the photon emission of methyl-substituted d-LH_2_ analogues 2b and 2c to that of the natural compound (2a) using an integrating sphere-based multichannel spectrometer with a charge-coupled device (CCD) detector (details are given in SI, Section S1.5). The photon/pixel emission spectra of the luciferase reaction showed that red-shifted methyl-substituted d-LH_2_ analogues (2b and 2c) provided greater photon emission than that of the natural compound (2a). Interestingly, 2b showed greater photon emission than those of 2a in Fluc, SLR, and Pmluc-N230S reactions (Fig. 4f–i). The spectra of 2b and 2c also showed the emission wavelengths longer than 600 nm. BL properties of d-LH_2_ analogues (2a–2c) correlate well with their quantum yields in the reactions of Fluc, SLR, and Pmluc-N230S measured later.

Importantly, the d-LH_2_ analogues (2b–2f) in this study allowed the reactions of Fluc and other luciferases to gain a maximum BL emission band shift (Δ^BL^*λ*) of >40 nm and up to 88 nm compared to 2a (Fig. 4j). The largest BL wavelength shift in this study could be obtained from the 2d-SLR orthogonal pair that emitted a maximum light peak at 663 nm and a broad BL spectrum beyond 750 nm, which could be beneficial for bio-imaging and real-time deep tissue *in vivo* monitoring applications in the future.

### Kinetics and thermodynamic properties of new d-LH_2_ analogues with different luciferases

To investigate the substrate specificity and the influence of substituents of d-LH_2_ analogues on BL characteristics, steady-state kinetics of luciferase (Fluc, SLR, Eluc, Pmluc-WT, and Pmluc-N230S) reactions using various d-LH_2_ analogues (2b–2f) as substrates were carried out at 25 °C and pH 8.0 to measure their kinetic constant, *K*_m_ and *k*_cat_, values compared to those of the natural d-LH_2_ (2a). The results in Table S6 show that, for all luciferases studied, the *K*_m_ values of 2b were comparable to those of 2a, suggesting that a methyl group substituent at the 5′-position of luciferin′s benzothiazole ring (2b) does not interfere with the ability of the enzyme to interact and react with a substrate. It should be noted that while the natural d-LH_2_ mostly exhibits strong substrate inhibition with a range of >1–10 µM against various luciferases, d-LH_2_ analogues (2b–2f) showed lower severity of substrate inhibition (Table S6). For the compounds with methoxy substituents at 5′ and 7′-positions or methyl substituents at the 4′ and 7′-positions, such as 2d, 2e and 2f, their *K*_m_ values were mostly greater than those of 2a, implying that these substituents may disrupt the interactions of these luciferins to serve as substrates for luciferases.

**Fig. 5:**
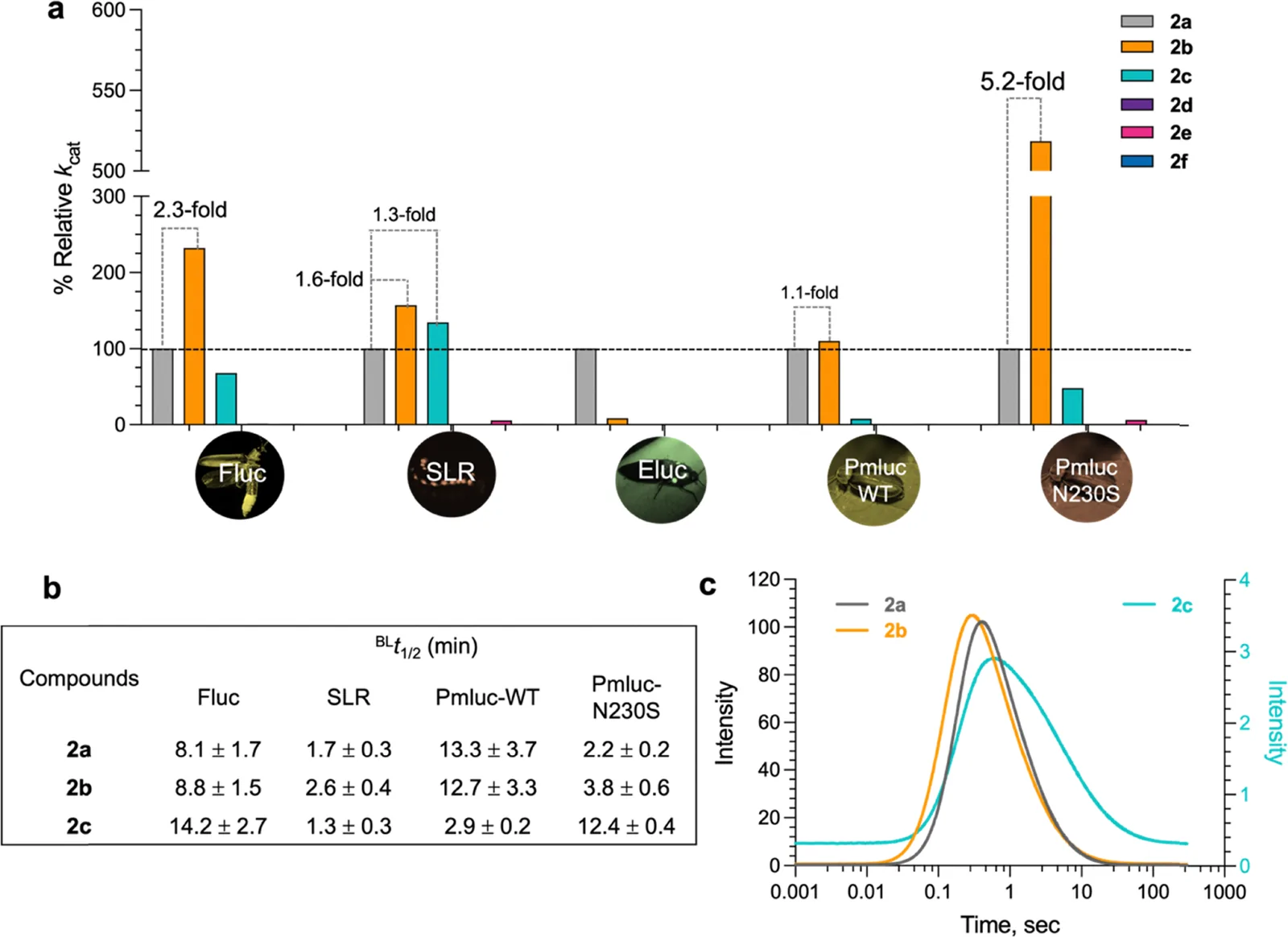
BL characteristics of d-LH_2_ analogues and luciferases. (a) The relative *k*_cat_, which represents the BL intensity (BLI) of each d-LH_2_ analogue (2a–2f) when it reacts with luciferases compared to those of 2a reactions. (b) The half-time (*t*_1/2_) values of BL emission of d-LH_2_ analogues (2a–2c) which react well with Fluc, SLR, Pmluc-WT, and Pmluc-N230S luciferases. (c) Kinetics of light formation and light decay of Fluc reactions with d-LH_2_ analogues (2a–2c) monitored by stopped-flow luminometry.

The *k*_cat_ values of the BL reactions indicate that the reactions of 2b with various luciferases, Fluc, SLR, Pmluc-WT, and Pmluc-N230S, yielded *k*_cat_ values greater than those of the native substrate (2a). In particular, the reaction with Pmluc-N230S resulted in a 5.2-fold increase in the *k*_cat_ value. However, the stability of the light emission (*t*_1/2_) of 2b is very similar to that of 2a in Fluc and Pmluc-WT, and light emission by SLR and Pmluc-N230S could be prolonged longer than those of other systems by about 1.5 and 1.7-fold, respectively (Fig. 5b). This result suggests that 2b with a methyl substituent at the 5′-position of the benzothiazole skeleton does not display any adverse effects on the activities of luciferases or their BL properties. The reactions of 2c exhibited *k*_cat_ values of approximately half that of 2a (except in the reaction of SLR, in which 2c gave a slightly higher *k*_cat_ than 2a by approximately 1.3-fold). 2d–2f provided very low bioluminescence intensity (BLI) in all luciferases tested (Fig. 5a and Table S7). The results indicate that 2d–2f showed larger *K*_m_ and lower BLI values than the native substrate (2a) in most of the enzyme reactions investigated. The data indicate that reactions of 2d–2f were not suitable with currently available luciferases and would require future work to engineer luciferases to obtain the best orthogonal pair of luciferase-d-LH_2_ analogues with high BLI. We noted that Eluc prefers to use 2a as a substrate rather than other analogues (Fig. 5a and Table S6) based on the *k*_cat_ and *K*_m_ values, but other enzymes such as Fluc, SLR, Pmluc-WT, and Pmluc-N230S could use 2b as an alternative substrate well. We also measured the stability of light emissions of the d-LH_2_ analogues (2a–2c). The results in Fig. 5b clearly indicate that 2c gave longer BL half-lives (*t*_1/2_) than the natural d-LH_2_ when using Fluc and Pmluc-N230S, by about 1.7- and 5.6-fold, respectively, as compared to those of 2a. Without the addition of coenzyme A (CoA), the ability of 2c in prolonging BL in Fluc and Pmluc-N230S should be useful for future live-cell imaging applications. Thus, the current best substrate analogues of luciferases such as Fluc, SLR, Pmluc-WT, and Pmluc-N230S are 2b and 2c which are also promising substrates for red-shifted BL and steady light emission in the reaction of SLR. Although 2d and 2f are novel compounds with potentially useful long wavelength emission, their usage with native luciferases is not very efficient due to their low BL signals. This is due to non-compatibility of these compounds with native enzymes. Engineering campaign of beetle luciferases is required for improving their BL signals in the future.

To comprehensively characterize the bioluminescence properties of the d-LH_2_ analogues (2a–2d), we measured their bioluminescence quantum yields from their reactions with Fluc, SLR, and Pmluc-N230S. The reactions were performed using the conditions obtained from steady-state kinetics studies (Fig. 5a and Table S6). Results shown in Table 1 and Table S8 indicate that the reaction of Fluc with 2b gave the greatest quantum yield (0.52) and the value was higher than those of the natural d-LH_2_ and other d-LH_2_ analogues. The data clearly support potential applications of Compound 2b. For the reactions with SLR and Pmluc-N230S, compounds 2a–2c showed the same range of quantum yields. However, the quantum yield of Compound 2d with all luciferases could not be measured due to low bioluminescence signals. Altogether, the results of quantum yield measurements indicate that the Fluc-2b pair gives the greatest value of BL signals.

**Table 1:** Quantum yields of luciferases and d-LH_2_ analogues

Luciferins	Quantum yield with different luciferases		
	Fluc	SLR	Pmluc-N230S
2a	0.44 ± 0.04	0.160 ± 0.005	0.160 ± 0.018
2b	0.52 ± 0.01	0.190 ± 0.005	0.170 ± 0.034
2c	0.200 ± 0.007	0.160 ± 0.003	0.150 ± 0.014
2d	ND^a^	ND^a^	ND^a^

a ND denotes “not determined” because 2d gave low bioluminescence signals.

Furthermore, the equilibrium binding constant (*K*_d_) values of d-LH_2_ analogues (2b–2d) with Fluc were measured using isothermal titration calorimetry (ITC) and compared with the values of natural d-LH_2_ (2a). The results in Table 2, Table S9 and Fig. S7 reveal that the *K*_d_ value of 2b was about half that of 2a, indicating that 2b has a greater binding affinity to Fluc than 2a. The data suggest that the 5′-methyl substituent in 2b does not interfere with the binding interactions between the substrate and the Fluc pocket site but rather promotes a tighter binding. For other d-LH_2_ analogues, the results indicate that modification of the luciferin benzothiazole ring at the 5′- and 7′-positions with methyl (2c) and methoxy groups (2d) yielded *K*_d_ values comparable to that of 2a, indicating that these substituents also do not interfere with the binding behavior. To investigate whether the pre-binding of ATP can enhance the interactions of d-LH_2_ analogues (2b–2d) with Fluc, we measured *K*_d_ values for binding of d-LH_2_ analogues to the preformed complex of Fluc and adenosine-5′-[(*α*,*β*)-methyleno]triphosphate (ApCPP) (Table 2 and S9). ApCPP is an ATP analogue mimicking the adenylated-ATP intermediate structure which does not allow Fluc to proceed with catalysis. This Fluc-ApCPP complex thus allowed us to probe interactions between d-LH_2_ analogues and the Fluc-ATP complex. Results in Table S9 indicate that all binding of d-LH_2_ analogues exhibited lower *K*_d_ values in the Fluc-ApCPP complex, implying that the presence of ATP enhances stronger interactions of d-LH_2_ analogues with Fluc.^30^

**Table 2:** Thermodynamics of ligand binding and kinetics constants for light formation and decay of the reactions of d-LH_2_ analogues (2a–2c) with Fluc

Compound	Dissociation constant (*K*_d_, µM)		Observed rate constant (*k*_obs_)		
	Fluc: Luciferin^a^	Fluc-ApCPP: Luciferin^b^	Light formation (s^−1^)	Light decay (s^−1^)	
				Faster phase	Slower phase
2a	21.1 ± 7.4	4.2 ± 0.1	6.64 ± 0.05	1.57 ± 0.02	0.29 ± 0.00
2b	13.4 ± 2.5	4.00 ± 0.02	9.63 ± 0.04	1.55 ± 0.15	0.27 ± 0.00
2c	35.1 ± 3.1	25.3 ± 1.1	7.28 ± 0.02	0.27 ± 0.00	0.05 ± 0.00
2d	30.4 ± 8.2	—c	8.13 ± 0.08	0.50 ± 0.00	ND^d^
2e	NC^e^	864 ± 7	—c	—c	—c

a The *K*_d_ values were measured by titration between Fluc and luciferins.

b The *K*_d_ values were measured by titration between Fluc-ApCPP complex and luciferins. To probe interactions between d-LH_2_ analogues and the Fluc-ATP complex, we used the non-hydrolyzable ATP analogue (ApCPP, Adenosine-5′-[(*α*,*β*)-methyleno]triphosphate) instead of ATP.

c —Denotes “not measured”.

d ND denotes “not determined” because 2d kinetics gave only one decay phase.

e NC denotes “not calculated” by ITC because 2e gave a very high *K*_d_ value with Fluc.

We also performed stopped-flow experiments to determine the observed rate constants (*k*_obs_) of single turnover reactions of light formation and decay of the Fluc reactions with 2a–2c (Fig. 5c and Table 2). The BL kinetics revealed three exponential phases (Fig. S6). The first phase was light formation, while the second and third phases were light decay with faster and slower rate constants, respectively. The kinetic analysis showed that all compounds displayed comparable *k*_obs_ values of light formation, suggesting that the substituents of d-LH_2_ analogues synthesized in this work do not affect the light formation kinetics. Interestingly, the *k*_obs_ values of light decay in both faster and slower phases of the 2c reaction were about 5–6-fold slower than those of 2a and 2b, indicating that the overall light emission yield in the reaction with 2c would be much greater than those of 2a and 2b.

We then used molecular docking and molecular dynamics (MD) simulations to explain the binding and kinetic behaviors of the d-LH_2_ analogues and luciferases as shown in Fig. S8–S19. The data revealed that all d-LH_2_ analogues (2b–2e) can bind to the active site of Fluc (Fig. S8a–e). To further investigate the stability of the enzyme and d-LH_2_ analogue complexes, MD simulations were performed across a range of temperatures (300–360 K). Results in Fig. S9–S18 showed that the structure of Fluc with 2a and 2b bound remained stable across all temperatures tested, particularly at the elevated temperature of 360 K compared to that of Fluc with 2c bound. These findings suggest that 2a and 2b exhibit stronger binding to Fluc compared to 2c; these data correlate well with *K*_d_ values obtained from the ITC experiments (Table 2).

In order to explain the equilibrium binding constant (*K*_d_) values of d-LH_2_ analogues (2b–2d) with Fluc, the crystal structure of the luciferase enzyme (Fluc) from *Photinus pyralis* with adenylate analogue (DLSA) bound (PDB: 4G36) was analyzed. Residues within 6 Å of the 7′-position of the bound DLSA are shown in Fig. S19. The 7′-position of DLSA is surrounded by the helix (F247 and T251), the lower beta strand (L286), the middle beta strand (A313 and S314), the middle loop (G315 and G316), and two upper beta strands (Q338, G339, Y340 and A348, L350, I351) which are linked by the top loop (S347). The introduction of a methyl substituent at the 5′-position could interact with T251, leading to the lower *K*_d_ of 2b compared to 2a. In contrast, the introduction of a methyl substituent at the 7′-position could clash with the middle beta strand (A313 and S314) and the upper beta strand (Q338, G339, Y340) leading to the higher *K*_d_ of 2c and 2d compared to 2a.

The overall data reported here indicate that 2b and 2c can be used well as dropped-in substrates for the currently available luciferases without requiring any enzyme engineering to provide red- shifted, high BL signals, and good light stability which could be useful for live-cell and animal BL applications in the future.

### Demonstration of the use of d-LH_2_ analogues in Mammalian cell assays

**Fig. 6:**
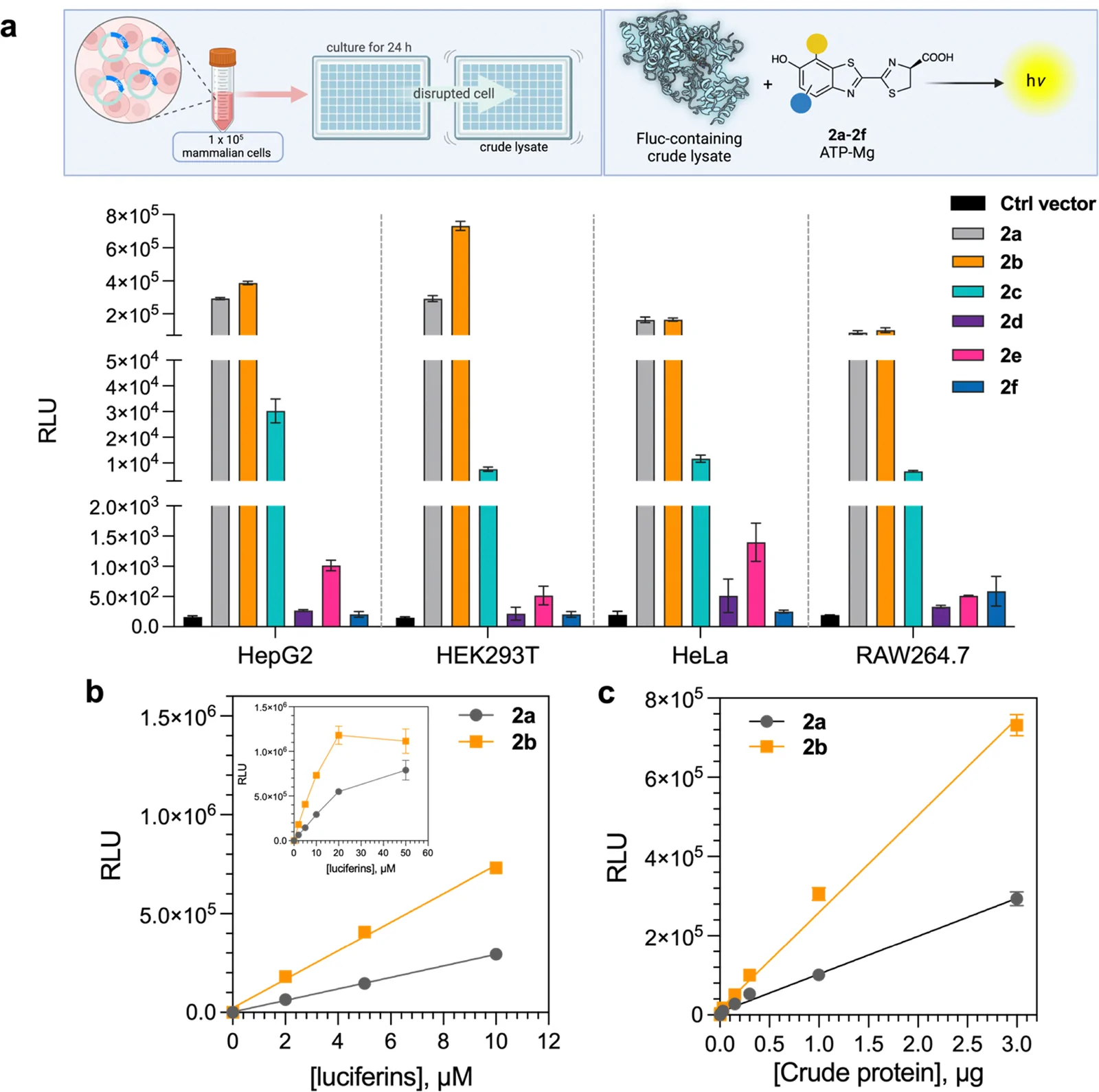
Applications of d-LH_2_ analogues (2a–2f) in mammalian cell line BL assays. (a) The crude lysate of each cell line was tested with d-LH_2_ analogues (2a–2f). (b) Comparison of light intensity from reactions of the Fluc-containing HEK293T crude lysate with 5′-MeLH_2_ (2b) and natural d-LH_2_ (2a) at various concentrations. (c) Dependence of light intensity on various concentrations of natural d-LH_2_ (2a) and 5′-MeLH_2_ (2b).

We tested the ability of the purified d-LH_2_ analogues (2a–2f) to serve as substrates for Fluc expressed in mammalian cell lines including HepG2, HEK293T, HeLa, and RAW264.7. These cells are commonly used as hosts for monitoring the gene expression, cellular activities and molecular reactions of diseases.^8,31–34^ BL signals generated by expression of Fluc in these cells have been used as tools to address various research topics in biomedical research. We transfected mammalian cells with the plasmid pGL4.13 expressing Fluc (details are given in SI, Section S1.6). Cells were collected after 24 h of growth for lysis, and the crude lysates were then assayed using the d-LH_2_ analogue cocktail reagents (2a–2f). The results showed that 2b provided light intensities similar to those of 2a with the crude lysates of HepG2, HeLa, and RAW264.7 (Fig. 6a). Interestingly, 2b showed a higher light intensity in the HEK293T crude lysate than 2a. In addition, 2c exhibited good light intensity with HepG2, HEK293T, HeLa, and RAW264.7 cell lysates, whereas other analogues (2d–2f) gave poor light intensity (Fig. 6a). All results suggested that 2b has good potential to be used as a substrate to generate red-shifted signals in BL applications because it can be used directly in commonly used cell-based assays with the wild-type Fluc.

We then further investigated the BL properties of 2b by varying its concentration in the reaction with a fixed amount of HEK293T crude lysate and compared the light intensity with that produced by the native substrate 2a. The results in Fig. 6b clearly demonstrated that 2b gave greater light intensity than 2a by about 2.5-fold. We then varied the concentrations of 2a and 2b with a fixed amount of HEK293T lysate and then varied the cell lysate amount with a fixed concentration of both compounds. The results as shown in Fig. 6b and c indicate that 2b provided greater sensitivity than 2a by about 2-fold.

The limit of detection (LOD) of expressed Fluc in crude cell lysates using 2a and 2b was identified to be 51.6 and 4.6 ng, respectively, indicating that 2b can give a better sensitivity than the natural d-LH_2_ (2a) by about 11.2-fold. Altogether, these results also confirmed that 5′-MeLH_2_ (2b) can serve as a promising substrate for future cell-based assay applications.

### Bioluminescence characteristics of d-LH_2_ analogues in live cell applications

**Fig. 7:**
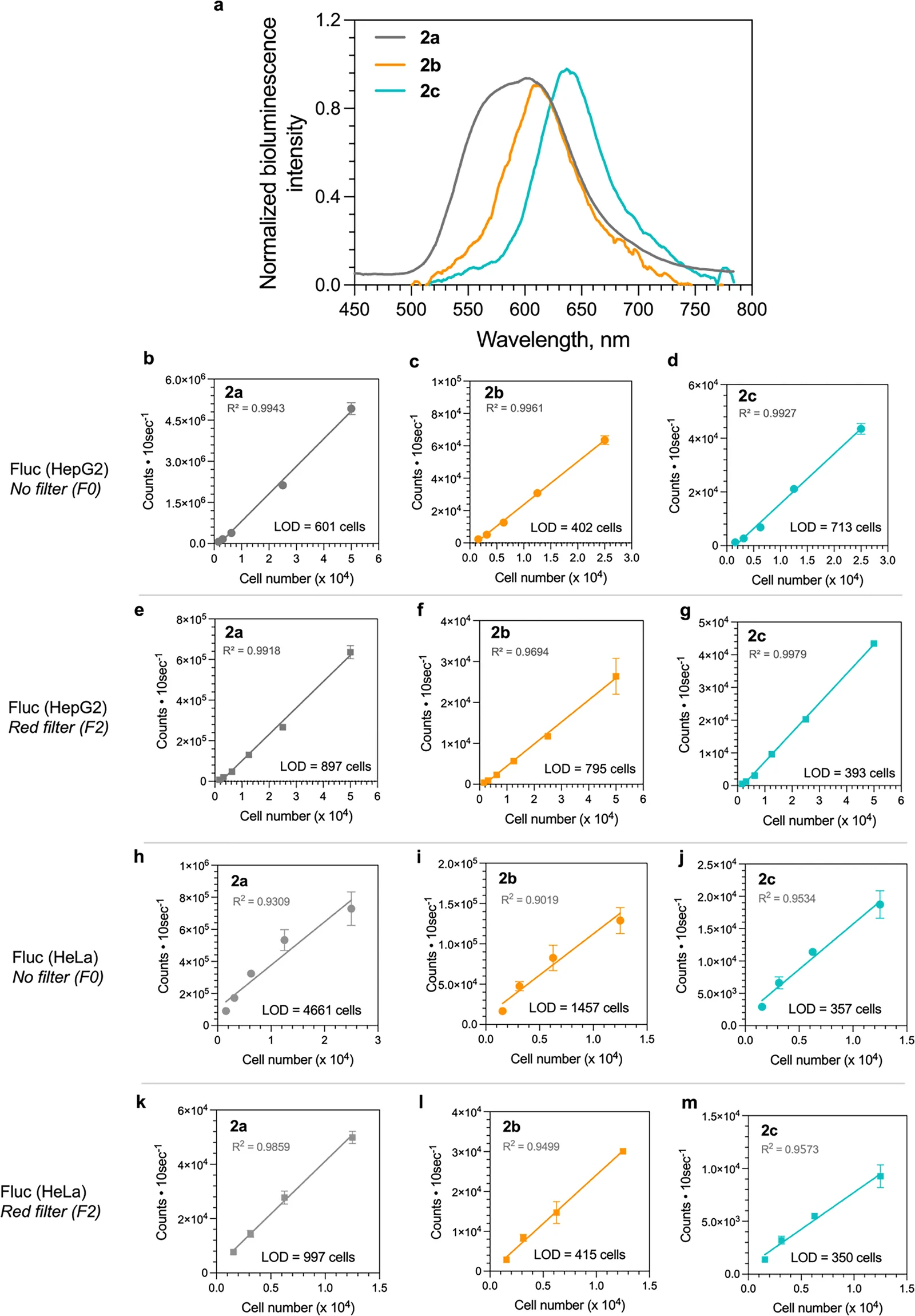
BL characteristics and applications of d-LH_2_ analogues (2a–2c) in live cells. (a) The BL spectra of Fluc and d-LH_2_ analogues (2a–2c) in stable reporter HepG2 cell lines harboring the fluc gene. The spectra were measured in live cells in 25 mM HEPES-NaOH pH 7.0 containing DMEM culture medium at 37 °C. The use of d-LH_2_ analogues (2a–2c) in living HepG2 cells. d-LH_2_ analogues were tested for their detection sensitivities by varying the number of HepG2 reporter cells using different BL filters including F0 (no filter) and F2 (red filter, >620 nm long-pass filter). (b–d) Linearity ranges of HepG2 cell number detection using 2a, 2b, and 2c with the F0 filter. (e–g) Linearity ranges of HepG2 cell number detection using 2a, 2b, and 2c with the F2 filter. The detection sensitivities of HeLa cells expressing fluc gene by transient transfection were tested with d-LH_2_ analogues using different BL filters including F0 (no filter) and F2 (red filter, >620 nm long-pass filter). (h–j) Linearity ranges of HeLa cell number detection using 2a, 2b, and 2c with the F0 (no filter). (k–m) Linearity ranges of HeLa cell number detection using 2a, 2b, and 2c with the F2 filter.

To demonstrate the use of d-LH_2_ analogues (2a–2c) in live cells, we measured the BL spectra of 2a–2c in the HepG2 cell line stably expressing a Fluc reporter (details are given in SI, Section S1.6). The BL spectra in intact cells were captured using a highly sensitive CCD spectrophotometer with 2 min exposure time. The spectra in Fig. 7a and Fig. S21 showed a broad spectrum of 2a-Fluc in live cells with a peak at 604 nm, which differed from those of the purified Fluc reactions (Fig. 4f). However, the spectra of 2b and 2c in living cells were identical to those of the purified Fluc reactions (refer to Fig. 7a and 4f) with red-shifted wavelengths at 609 and 638 nm, respectively. HepG2 and HeLa cell lines were chosen for further investigation because they are among the most commonly used cell lines in biomedical research (41 200 and 46 000 publications in 2023, respectively, searched by Google Scholar, Fig. S20).

We further investigated the sensitivity of reporter HepG2 and HeLa cell line detection when 2a–2c were used as substrates. The number of HepG2 and HeLa cell lines was varied by 2-fold dilution and the cells were seeded in a 96-well plate. 100 µM (final concentration) of 2a–2c in culture medium was then added to the cells and incubated for 30 min. The BL signals (photon counts) after a 30 min incubation period were measured with different filters to increase the selectivity for the BL wavelength—especially towards red-shifted light. In this work, we used optical filters including F0 (no filter) and F2 (red filter, >620 nm long-pass filter) to enhance the BL signals at specific wavelength regions to reduce background signals. The detection of live HepG2 and HeLa cell lines using an F0 filter (no filter) in Fig. 7b-m showed a good linearity (*R*^2^ > 0.99) with all compounds (2a–2c). The sensitivity for detection in live Fluc-expressing HepG2 cells using 2b was better than those of 2a and 2c, with an LOD of approximately 402 cells in the assay, using the F0 filter (no filter). The results in Fig. 7e–g showed linearity ranges for detection by 2a–2b measured using the F2 filter, which collected light signals >620 nm. 2c showed a prominent ability of red emitted signals for live cell detection in red BL regions, displaying high sensitivity when measured in HepG2 cells with a red filter, which required only 393 cells. In contrast, LODs for the similar systems with 2a and 2b are 897 and 795 cells, respectively (Table 3).

**Table 3:** Detection sensitivities of luciferase-expressing cells treated with d-LH_2_ analogues using different optical filters

Reporter cell	Filter	Limit of detection (LOD), cells		
		2a	2b	2c
Fluc (HepG2)	F0	601	402	713
SLR (HepG2)		802	643	500
Pmluc-N230S (HepG2)		2010	752	ND^a^

Fluc (HepG2)	F2	897	795	393
SLR (HepG2)		1071	783	613
Pmluc-N230S (HepG2)		1880	247	ND^a^

Fluc (HeLa)	F0	4661	1457	357
SLR (HeLa)		1352	973	1437
Pmluc-N230S (HeLa)		1428	630	ND^a^

Fluc (HeLa)	F2	997	415	350
SLR (HeLa)		1268	826	707
Pmluc-N230S (HeLa)		1185	812	ND^a^

a ND denotes “not determined” because 2c gave weak BL signals.

Interestingly, the sensitivity for detection in live Fluc-expressing HeLa cells treated with 2c gave an LOD of approximately 357 cells which had higher sensitivity than those of 2b and 2a about 4.1 and 13.0-fold, respectively, using the F0 filter (no filter) (Fig. 7h–j and Table 3). Detection using the F2 filter for HeLa reporter cells also showed the LODs with 2c, 2b, and 2a as 350, 415, and 997 cells, respectively (Fig. 7k–m and Table 3). The HeLa cells with 2c gave higher sensitivity than that of 2a about 2.8-fold using the F2 filter.

We further tested the usage of d-LH_2_ analogues (2a–2c) with SLR and Pmluc-N230S expressing HepG2 and HeLa cells. Results in Fig. S22 and S23 and Table 3 indicate that most of the red-shifted d-LH_2_ (2b and 2c) provided higher sensitivity with lower LOD values than that of 2a, except for Pmluc-N230S-expressing HepG2 and HeLa cells treated with 2c.

The results suggested that methyl-substituted luciferins (2b and 2c) can permeate through the cell membrane of live cells and serve as substrates for Fluc inside the cytosol well. The high light intensity of 2b in this red BL region also demonstrated its ability to serve as a new substrate (in replacement of 2a) for luciferases. 2c also showed high BL signals at longer red-shifted wavelengths which was suitable for detecting luciferase-expressing cells in low numbers because it provides high sensitivity for red light emission.

**Fig. 8:**
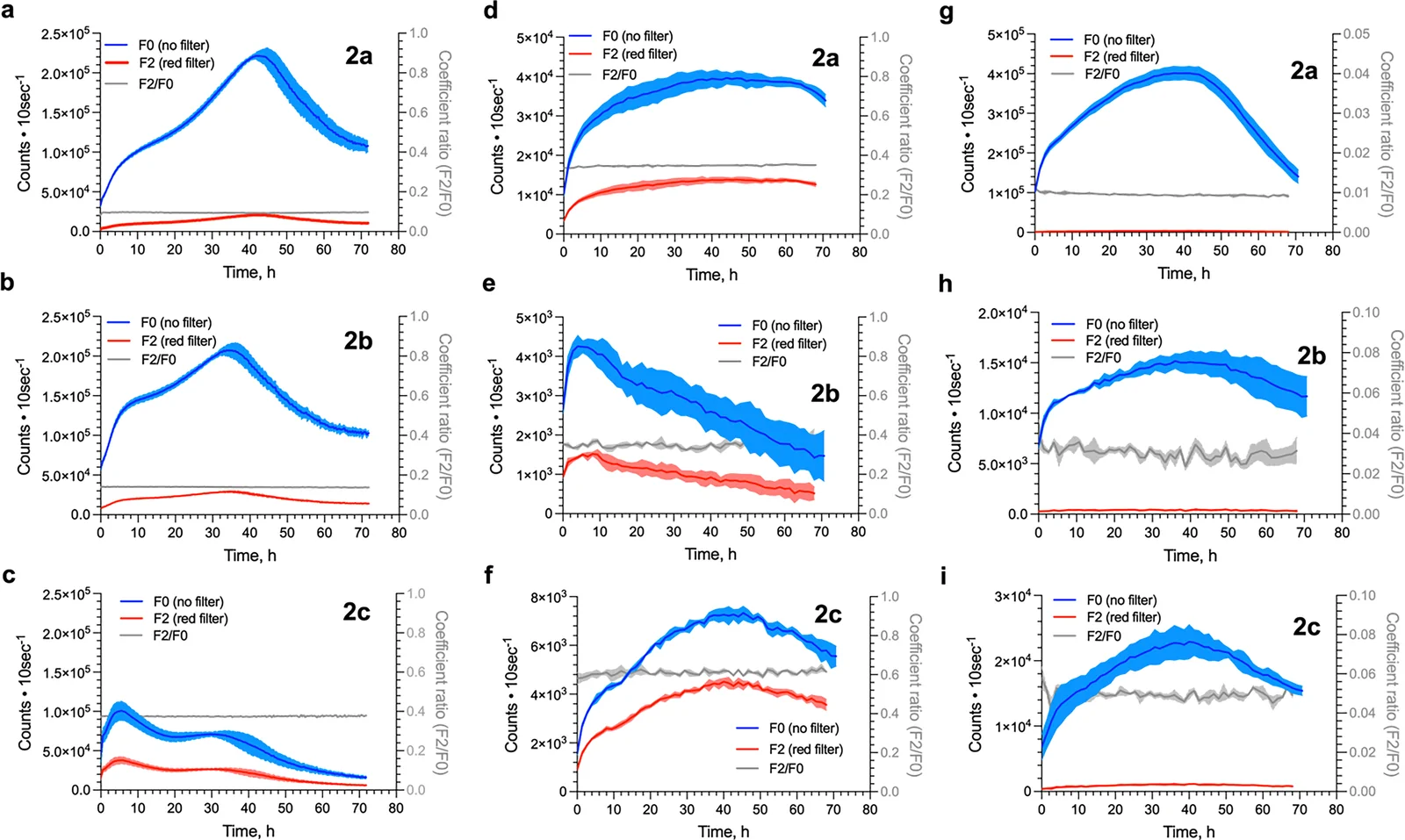
Real-time monitoring of BL signals of HepG2 cell lines with luciferase reporters and d-LH_2_ analogues (2a–2c). (a–c) BL real-time monitoring signals of stably Fluc-expressing HepG2 cells with natural d-LH_2_ (2a), 5′-MeLH_2_ (2b), and 5′,7′-DiMeLH_2_ (2c), respectively. (d-f) BL real-time monitoring signals of transient transfection of SLR reporter in HepG2 cells with natural d-LH_2_ (2a), 5′-MeLH_2_ (2b), and 5′,7′-DiMeLH_2_ (2c), respectively. (g–i) BL real-time monitoring signals of transient transfection of Pmluc-N230S reporter in HepG2 cells with natural d-LH_2_ (2a), 5′-MeLH_2_ (2b), and 5′,7′-DiMeLH_2_ (2c), respectively.

The reporter HepG2 cell line expressing Fluc, SLR, and Pmluc-N230S was also real-time monitored after adding 100 µM (final concentration) of d-LH_2_ analogues (2a–2c) in Fig. 8a-i. The BL characteristics of all d-LH_2_ analogues in live cells were measured for 3 days. The results showed that the light intensity of 2b in stable Fluc-expressing reporter cells was slightly higher than that of 2a for 24 h when measured using both F0 and F2 filters and then gradually decreased over time after 30 h. 2c showed greater red BL signals (highest F2/F0 ratio) than other compounds but gave lower BL intensity than 2a and 2b when using the F0 filter or no filter was used (Fig. 8a–c). The highest BL signals of 2a–2c could be achieved at 44, 35, and 6 h, respectively. The data indicate that the highest signals of 2c and 2b BL could be obtained at 7.3 and 1.2-fold faster rates than that of 2a, respectively. This may be due to the ability of 2b and 2c to penetrate the cells faster than 2a.

The transient SLR-expressing HepG2 cells with 2b showed faster light generation kinetics, which gradually decreased after 10 h. The real-time BL signal patterns of 2a and 2c were also similar to that of 2b, with 2c exhibiting the highest F2/F0 ratio (red BL characteristics) than those of 2a and 2b (Fig. 8d–f). The transient Pmluc-N230S-expressing HepG2 cells showed similar kinetic patterns with all compounds 2a–2c (Fig. 8g–i). The cells with 2b and 2c appeared to prolong the BL signals after 40 h better than that of 2a.

Moreover, when the HeLa cell lines transiently expressing Fluc, SLR, and Pmluc-N230S were treated with 100 µM (as a final concentration) of d-LH_2_ analogues (2a–2c), their real-time monitored kinetic characteristics were different from those of HepG2 cells (Fig. S24). The highest BL signals could be achieved approximately at 510 h. After a maximum peak at 5 h, the BL signal of the 2a compound (a native d-LH_2_) with Fluc-expressing HeLa cells gradually decreased. BL signals of 2b and 2c could be prolonged much better, up to 13 h before decreasing (Fig. S24b and c). Similarly, the kinetics patterns in SLR-expressing HeLa cells (Fig. S23d–f) showed more prolonged BL signals (up to 18 h) with 2c than those of 2a and 2b compounds (up to 10 h) before signal decreasing. The Pmluc-N230S-expressing HeLa cells exhibited low BL signals with all 2a–2c compounds, particularly with the 2c compound (Fig. S23g and h). Kinetics of these BL signals indicate the ability of these d-LH_2_ analogues (2a–2c) to penetrate the cell membrane and maintain their activities inside the cells.

All results suggest that d-LH_2_ analogues with a methyl substituent at the benzothiazole ring, including 2c and 2b, can penetrate the cells faster than natural d-LH_2_ (2a). Therefore, 2b and 2c showed great potential to be used as substrates for cell-based assays and real-time monitoring experiments.

## Conclusions

Here, we developed a new setup for cost-effective one-pot green synthesis of six d-LH_2_ analogues using mild chemical reagents. We discovered that by establishing a continuous feeding of d-cys (2 eq.) into an anaerobic one-pot reaction containing anaerobic 1a (1 eq.), the substrate instability (1a derivatives and d-Cys) and yield of d-LH_2_ analogues could be significantly improved. The process could improve the production yield by 63-fold compared to the previously reported chemical condensation method.^17^ The synthesis method here could synthesize two new d-LH_2_ analogues including 5′,7′-DiMeOLH_2_ (2d) and 7′-MeNpLH_2_ (2f). Substituents such as methyl, methoxy, and naphthol groups at the luciferin′s benzothiazole ring (2b–2f) generated longer red-shifted BL wavelength than that of the natural d-LH_2_ (2a) by up to 88 nm when reacting with five types of beetle luciferases. The longest red-shifted BL could be obtained from the 2d-SLR orthogonal pair reaction with a maximum BL at 663 nm. However, the reactions of luciferases with 2d and 2f gave low signals; this requires enzyme engineering to improve the BL signals of 2d and 2f in the future.

We demonstrated the use of d-LH_2_ analogues (2a–2f) in mammalian cell lines. The crude lysate assays exhibited good light intensity in the reactions of 2b and 2c in HepG2, HEK293T, HeLa, and RAW264.7 cells, especially 2b provided greater detection sensitivity of the Fluc enzyme in crude lysates than 2a by about 11.2-fold; this is beneficial for bio-reporter and cell-based assay applications. Real-time BL measurements in live cells showed that 2b and 2c provide greater BL red-shifted signals than 2a. Altogether, our results indicate that a small methyl group substituent in the luciferin′s benzothiazole ring does not disrupt the activity of wild-type beetle luciferases but enhances light intensity, red-shifted BL, and provided greater light stability—features that are highly advantageous for *in vitro*, *ex vivo*, and *in vivo* BL detection applications in the future.

## Author contributions

P. W. and P. C. designed experiments; P. W., I. K., P. K., C. K., and D. S. performed experiments. I. K., W. J. and P. W. elucidated the structures of synthesized d-LH_2_ analogues by QTOF-MS and NMR. P. W. and C. K. optimized the conditions of bioluminescence assays. P. W. characterized the synthesized d-LH_2_ analogues. N. L. and A. P. performed molecular docking and MD simulations. P. W., I. K., P. K., W. J., C. K., D. S., A. P., N. L., S. V., R. T., S. M., J. S., Y. N. and P. C. performed the data analysis. P. C., R. T., S. M., J. S., R. N., K. N., Y. N., and Y. O. provided advice. P. W. and P. C. wrote the manuscript with suggestions and comments from all authors.

## Conflicts of interest

A patent application related to synthesis of red-shifted luciferin analogues has been submitted to the Department of Intellectual Property, Thailand (TH 2301002330).

## Supplementary Material

CB-007-D5CB00287G-s001

## Data Availability

The authors declare that all data supporting the findings of this study are available within the paper and its supplementary information (SI). Supplementary information: experimental procedures, tables and figures showing d-LH_2_ analogue synthesis, yield optimization, structural elucidation and bioluminescence characterization, and molecular docking, and applications of d-LH_2_ analogues in crude lysates and live mammalian cell lines. See DOI: https://doi.org/10.1039/d5cb00287g.
