# Supplementary material for: Red-shifted d-luciferin analogues and their bioluminescence characteristics
Source: RSC Chem Biol. 2025 Nov 14;7(1):67–80. doi: 10.1039/d5cb00287g (PMC12668289; doi:10.1039/d5cb00287g)
Supplement: CB-007-D5CB00287G-s001 [file CB-007-D5CB00287G-s001.pdf]

Supplementary Information (SI) for RSC Chemical Biology.
This journal is © The Royal Society of Chemistry 2025

Supplementary Information

Red-Shifted D-Luciferin Analogues and their Bioluminescence Characteristics

Pratchaya Watthaisong,[a] Chadaporn Kantiwiriyawanitch,[a] Watcharapa Jitkaroon,[a] Aisaraphon Phintha,[a] Ittiphat Klayparn,[a] Narin Lawan,[b] Philaiwarong Kamutira,[a] Daisuke Sasaki,[c] Surawit Visitsatthawong,[a] Somchart Maenpuen,[d] Ruchanok Tinikul,[e] Jeerus Sucharitakul,[f] Ryo Nishihara,[g] Kazuki Niwa,[h] Yoshihiro Nakajima,[c] Yoshihiro Ohmiya,[i] and Pimchai Chaiyen*[a]

[a.] Biomolecular Science and Engineering (BSE), Vidyasirimedhi Institute of Science and Technology (VISTEC), Wangchan Valley, Rayong, 21210, Thailand,

[b.] Department of Chemistry, Faculty of Science, Chiang Mai University, Chiang Mai, Thailand

[c.] Health and Medical Research Institute, National Institute of Advanced Industrial Science and Technology (AIST), Takamatsu, Kagawa 761-9035, Japan

[d.] Department of Biochemistry, Faculty of Science, Burapha University, Bangsaen, Chon Buri District, Chon Buri 20131 Thailand

[e.] Department of Biochemistry, Faculty of Science, Mahidol University, Ratchathewi, Bangkok 10400 Thailand

[f.] Department of Biochemistry, Faculty of Science, Chulalongkorn University, Pathum Wan, Bangkok 10330 Thailand.

[g.] Health and Medical Research Institute, National Institute of Advanced Industrial Science and Technology (AIST), Tsukuba, Ibaraki 305-8566, Japan

[h.] National Metrology Institute of Japan, National Institute of Advanced Industrial Science and Technology (AIST), Tsukuba, Ibaraki 305-8563, Japan

[i.] Biomedical Research Institute, National Institute of Advanced Industrial Science and Technology (AIST), Ikeda, Osaka 563-8577, Japan

*Correspondence: pimchai.chaiyen@vistec.ac.th

Table of Contents

1. Experimental Procedures 4

1.1 Synthesis of firefly luciferin (D-LH_2) and analogues 4

1.2 Reaction optimization to increase yield of D-LH_2 formation 4

1.3 A setup of substrate continuous feeding to improve the yield of D-LH_2 4

Experimental Table 1 4

1.4 Purification of D-LH_2 analogues and their structure elucidation 5

1.5 Bioluminescence characterizations 5

1.6 Mammalian cell line culture and bioluminescence assays 6

1.7 Molecular docking of luciferases and luciferins 7

1.8 Molecular docking and molecular dynamics (MD) simulations of luciferase and luciferin complexes 7

2. Supplementary Information 7

2.1 Identification of firefly luciferin (D-LH_2) and analogues

7

Supplementary Table S1 8

2.2 Effects of buffer type on D-LH_2 synthesis 8

Supplementary Fig. S1 8

2.3 Effects of co-solvent on D-LH_2 formation 9

Supplementary Fig. S2 9

2.4 New firefly luciferins and their structural elucidation 9

Supplementary Fig. S3 10

Supplementary Table S2 10

Supplementary Fig. S4 11

Supplementary Table S3 11

2.5 Effects of oxygen on D-LH_2 formation 12

Supplementary Fig. S5 12

Supplementary Table S4 12

2.6 Spectra of firefly luciferins 13

Supplementary Table S5 13

2.7 Steady-state kinetics of the reactions of luciferases and D-LH_2 analogues 13

Supplementary Table S6 14

2.8 Investigation of bioluminescence kinetics using stopped-flow luminometry 15

Supplementary Fig. S6 15

Supplementary Table S7 15

2.9 Measurement of quantum yield values 15

Supplementary Table S8 16

2.10 Measurement of K_d and thermodynamics parameters by ITC 16

Supplementary Fig. S7 16

Supplementary Table S9 17

2.11 Molecular docking and molecular dynamics simulations of beetle luciferase and D-LH_2 analogues 17

2.11.1 Molecular docking of Fluc with **2a**-**2e** 18

Supplementary Fig. S8 18

2.11.2 Molecular dynamics (MD) simulation of Fluc with **2a**-**2e** 18

Supplementary Fig. S9 19

Supplementary Fig. S10 20

Supplementary Fig. S11 20

Supplementary Fig. S12 21
Supplementary Fig. S13 21
Supplementary Fig. S14 22
Supplementary Fig. S15 22
Supplementary Fig. S16 23
Supplementary Fig. S17 24
Supplementary Fig. S18 25
Supplementary Fig. S19 26
2.12 Comparison of cell line usage in research 26
Supplementary Fig. S20 26
2.13 Bioluminescence spectra of live HepG2 cells 27
Supplementary Fig. S21 27
2.14 Sensitivity of reporter cells detection 27
Supplementary Fig. S22 28
Supplementary Fig. S23 29
2.15 Real-time monitoring of bioluminescence of HeLa reporter cells 30
Supplementary Fig. S24 30

1. Experimental Procedures

1.1 Synthesis of firefly luciferin (D-LH_2) and analogues

Reactions for synthesis of D-LH_2 and D-LH_2 analogues were carried out in 5 ml of 100 mM HEPES-NaOH pH 8.0 containing 1 mM 1,4-benzoquinone (BQ) derivatives (**1a-1f**) and 2 mM D-cysteine (D-Cys). The reactions were shaken at 25 °C, 250 rpm for 24 h before they were quenched and diluted by adding methanol (MeOH). The resultant mixtures were centrifuged (13,800 × g) for 30 min to obtain a clear supernatant which was then filtered through a 0.45 µm nylon membrane filter. The filtrate (3 replications for each point of measurement) was subjected to analysis by HPLC/ESI-QTOF-MS (Bruker, Germany) in positive ionization mode to identify the products (**2a-2f**) formed.

1.2 Reaction optimization to increase yield of D-LH_2 formation

To improve the yield of D-LH_2 formation, we further explored effects of co-solvent and oxygen (O_2). The synthesis reaction was carried out in 5 ml of 100 mM HEPES-NaOH pH 8.0 containing 1 mM of **1a** and 2 mM D-Cys. Concentrations of co-solvents (ethanol (EtOH), MeOH, acetonitrile (ACN), (+)-limonene, and *p*-cymene) were varied from 0-50 % (v/v). The effect of O_2 on D-LH_2 formation was tested by varying the O_2 percentage in the reaction from 20% (ambient), 10%, and <0.0005% (anaerobic condition). The reaction was shaken at 25 °C, 250 rpm for 24 h. The reaction solution (0.5 ml) was taken, quenched and diluted by MeOH, then centrifuged (13,800 ×g) for 30 min. The clear supernatant was filtered through a 0.45 µm nylon membrane filter. The filtrate (3 replications for each point of measurement) was subjected to analysis by HPLC/triple quadruple (QQQ)-MS (positive mode) to determine the yield of D-LH_2 formed.

1.3 A setup of substrate continuous feeding to improve the yield of D-LH_2

Semi large-scale D-LH_2 production by a one-pot reaction was carried out in a 100-mL reaction. The reaction contained 20 mM and 40 mM D-Cys and 20% (v/v) MeOH in 100 mM HEPES-NaOH (pH 8.0) in a bottle under nitrogen atmosphere (anaerobic condition). Then, 10 mL of 100 mM *p*-benzoquinone (**1a,** final concentration is 10 mM) was constantly fed into the reaction bottle using a syringe pump (flow rate 2 ml/h) and stirred (250 rpm) at room temperature. The samples were collected at 12, 24 and 48 hours and quenching reagents were added as described in section 1.2. The sample was analyzed by HPLC-QQQ-MS (positive mode) to quantify the amount of D-LH_2 (see diagram in main text Fig. 3 and Table S1).

On another setup, a reaction bottle containing 10 mM, 20 mM and 40 mM of **1a** and 20% (v/v) MeOH in 100 mM HEPES-NaOH (pH 8.0) was continuously fed with 10 mL of 100 mM and 200 mM D-Cys using a syringe pump (2 ml/h). The sample preparation was prepared as mentioned above and subjected into HPLC-QQQ-MS (positive mode) analysis to quantify the amount of D-LH_2 (**2a**) (see diagram in main text Fig. 3 and Table S1).

Experimental Table 1. Conditions for setting up of substrate continuous feeding in one-pot reaction

Entry	Syringe		Bottle		Substrate ratio
	Substrate	Concentration, mM	Substrate	Concentration, mM	
1	BQ (**1a**)	10	D-Cys	20	1:2
2	BQ (**1a**)	10	D-Cys	40	1:4
3	D-Cys	10	BQ (**1a**)	20	1:2
4	D-Cys	10	BQ (**1a**)	40	1:4
5	D-Cys	20	BQ (**1a**)	10	2:1

1.4 Purification of D-LH_2 analogues and their structure elucidation

A semi large-scale one-pot reaction of D-LH_2 analogues (**2a-2f**) synthesis was carried out in a 100-ml reaction. The freshly prepared and O_2-free 10 mM BQs (**1a-1f**) and 20 mM D-Cys were mixed in 20 % (v/v) and 100 mM HEPES-NaOH pH 8.0 under nitrogen atmosphere. The mixture was stirred for 24 h at room temperature. The resultant solution was washed with ethyl acetate (50 ml x 1) and the aqueous layer was acidified with 12 M HCl to obtain pH below 3. The acidified solution was extracted with ethyl acetate (50 ml x 3). The combined organic layers were dried over anhydrous Na_2SO_4, filtered, and concentrated by a rotary evaporator (BUCHI, Rotavapor, R-100). The dried fraction was resuspended with MeOH and loaded onto a gel filtration column (Sephadex-LH20, GE Healthcare). MeOH was used as an eluent to elute the luciferin analogues out from the column. The dried fraction was further purified by anion-exchange chromatography (DEAE Sepharose). Each fraction was spotted on a RP-TLC plate and the TLC plates were developed with 50 % (v/v) of $MeOH/H_2O$. Spots of individual fractions were visualized by UV radiation to identify the luciferin derivatives for further characterizations by NMR spectroscopy. The yellow powder of luciferin analogues was dissolved in MeOH-d_4 (MeOD) and its ^{1}H- and ^{13}C-NMR spectra were recorded using a Bruker AVANCE III HD spectrometer (600 MHz).

We also carried out a semi large-scale synthesis of native D-LH_2 (**2a)** and Compound **2c** using the benzoquinone (BQ) derivatives, L-Cys methyl ester and D-Cys condensation protocol recently reported[1]. However, this protocol only worked well for preparation of Compounds **2a** and **2c** while for preparation of other D-LH_2 analogues (**2b, 2d-2f**), the protocol developed in our report here is the suitable one to use.

1.5 Bioluminescence characterizations

Reactions of luciferases, including *Photinus pyralis* luciferase (Fluc), *Phrixothrix hirtus* luciferase (SLR), *Pyrearinus termitilluminans* luciferase (Eluc), *Pyrocoelia miyako* luciferase (Pmluc) and Pmluc-N230S), with D-LH_2 analogues (**2a-2f**) were carried out by mixing 0.2 mM D-LH_2 analogues, 3 mM ATP, 6 mM $MgCl_2$, and 10 µM luciferase in 0.2 ml of 100 mM HEPES-NaOH pH 8.0. The bioluminescence (BL) emission characteristics were recorded using a microplate reader in a BL mode (Thermo Scientific, Varioskan, LUX multimode). A digital single-lens reflex (DSLR) camera (Nikon D80, CCD sensor) with a 30-second exposure period was used to capture the BL images. **2a**-**2c** were selected to investigate their BL emission spectra with luciferases including Fluc, SLR, Pmluc-WT, Pmluc-N230S using an integrating sphere-based multichannel spectrometer with a charge-coupled device (CCD) detector.

Stability of BL signals from luciferases (Fluc, SLR, Eluc, Pmluc-WT, Pmluc-N230S) and various D-LH_2 analogues (**2a-2f**) were tested and compared. The reactions were performed in 100 mM HEPES-NaOH pH 8.0 by mixing a solution (25 µl) of 10 µM D-LH_2 analogues (**2a-2f**) premixed with 70 nM Fluc solution with a cocktail solution (125 µl) of 3 mM ATP and 6 mM $MgCl_2$ to initiate the reaction. BL of the reaction at various time points was recorded by luminometer (Luminescencer-Octa, AB-2270, ATTO) and the light stability was compared.

Steady-state kinetics of luciferases (Fluc, SLR, Eluc, Pmluc-WT, Pmluc-N230S) using D-LH_2 analogues (**2a-2f**) as substrates were carried out in 100 mM HEPES-NaOH (pH 8.0) and monitored total light emission using a luminometer (Luminescencer-Octa, AB-2270, ATTO). Various concentrations of **2a-2f** were individually premixed with luciferases. The reactions were initiated by mixing 25 µl of premixed solution of luciferases/D-LH_2 analogues with 125 µl of 3 mM ATP and 6 mM $MgCl_2$ in 100 mM HEPES-NaOH (pH 8.0). The light intensity (relative light unit, RLU) during 0-300 sec was then collected to represent the overall luciferase activity which was further used for calculating kinetic parameters from the Michaelis-Menten equation.

Pre-steady-state kinetics of light formation and decay of Fluc reactions and D-LH_2 analogues (**2a-2d**) was investigated using a stopped-flow spectrophotometer (SF61-DX model, TgK Scientific). One syringe was filled with a solution of 50 µM Fluc, 3 mM ATP, and 6 mM $MgCl_2$ while another syringe was filled with a solution of 10 µM D-LH_2 analogues (**2a-2d**), both in 100 mM HEPES-NaOH (pH 8.0). Solutions in both syringes were mixed by a stopped-flow device and measured the light emission at 25 °C in triplicate. The kinetic traces were analyzed using Kinetic Studio software program (TgK Scientific) to analyze and calculate the observed rate constants (k_{obs}) of each kinetic phase.

We measured quantum yields (QY) of luciferases (Fluc, SLR, Pmluc-N230S) with D-LH_2 analogues (**2a-2d**) following the protocols previously reported[2, 3]. Solutions of purified luciferases including Fluc, SLR and Pmluc-N230S and D-LH_2 analogues (**2a-2d**) were prepared in 100 mM Tris-HCl pH 8.0. A reference light source was used for calibrating a sphere spectrometer equipped with a cooled CCD detector. The absolute responsivity of each luciferase-luciferin reaction was calculated based on a linear standardization between total photon flux measured using the sphere spectrometer equipped with a cooled CCD detector and relative count values measured by a luminometer. Total photons emitted from the bioluminescence reaction of each luciferase-luciferin pair were measured using a calibrated luminometer. A reaction containing 0.5-5 nM of each luciferin, 3 mM ATP, 3 mM $MgCl_2$ in 100 mM Tris-HCl (total volume of 200 µl) was placed into the luminometer and background signals were recorded. The reaction was then initiated by adding 20 µl of 0.5

mM purified luciferases into the reaction tube and the bioluminescence signals were monitored until it decayed to the background level. The QY value was calculated from the measured total numbers of photon emission from the reaction of each D-LH_2 analogue with luciferase.

1.6 Mammalian cell line culture and bioluminescence assays

Human embryotic kidney (HEK293T) cells, human hepatocyte carcinoma cancer (HepG2) cells, human cervical carcinoma (HeLa) cells, and Abelson leukemia virus-transformed cell line derived from BALB/c mice (RAW264.7) cells (American Type Culture Collection, USA) were grown in Dulbecco's Modified Eagle Medium (DMEM) in low glucose supplemented with 10% (v/v) heat-inactivated fetal bovine serum (FBS) plus 1% (w/v) penicillin-streptomycin. Four types of cells were maintained at 37 °C with 5% CO_2. For transient transfection, cells were seeded in 24-well plates at a density of $1×10^5$ cells per well and allowed to attach overnight in the DMEM medium supplemented with 10% (v/v) heat-inactivated FBS. Before transfection, the cultured medium was removed and added a fresh minimal essential medium with reduced amount of serum (opti-MEM™) to each well. Cells were transfected by adding 0.07 pmol of pGL4.13 [*luc*2/SV40] as a target vector and 0.007 pmol of pRL-TK as a control vector using lipofectamine™3000 (Thermo Fisher Scientific) in opti-MEM™ and lipofection. Cells were incubated for 5 h and then the supernatant medium was replaced by a fresh working DMEM medium. The transfected cells were maintained for 24-h. Cells were harvested by washing with 500 µl PBS buffer (pH 7.4). The cells were detached and collected by adding 200 µl 1x lysis buffer reagent and subsequently rocking at room temperature for 15 min. Cells were lysed by a freezing-thawing process. The cell lysate was collected by centrifugation (13,800 × g) at 4 °C for 10 min and the supernatant was then kept at -80 °C until used.

Activities of Fluc with D-LH_2 analogues (**2a-2f**) were measured by analyzing the BL signals (RLU) for 30 sec using a luminometer (Luminescencer-Octa, AB-2270, ATTO). The 150 µl assay reaction was carried out in 150 µl 100 mM HEPES-NaOH (pH 8.0). The 25 µl premixed solution of cell line lysate/D-LH_2 analogues (**2a-2f**) was added 125 µl of 3 mM ATP and 6 mM $MgCl_2$ to initiate the reaction in 100 mM HEPES-NaOH (pH 8.0). Then, the RLU signals of each D-LH_2 and analogues were compared.

To compare light intensity (RLU), **2a** and **2b** were selected for further investigation with HEK293T crude lysate (6.0 µl, ~0.1 µg of total proteins) and a substrate cocktail reagent (3 mM ATP and 6 mM $MgCl_2$ in 100 mM HEPES pH 8.0). The detection sensitivity of crude lysate of HEK293T expressing Fluc was determined by measuring RLU of the reaction with a luminometer with concentrations under the linearity range (10 µM) of **2a** and **2b**.

To make an expression plasmid carrying *fluc* gene, the *luc2* gene containing SV40 poly A signal in the pGL4 plasmid (Promega) (pGL4.10[*luc2*]) was digested with *Hind*III and *Bam*HI and ligated into the HindIII/*Bam*HI site of pCAG-GLuc in which GLuc was removed[4]. The resulting plasmid was recombined into the pφC31-Neo attB[5] vector as reported previously[4, 6], resulting in the pCAG-luc2-φC31-Neo.

To generate a stable HepG2 cell line, the pCAG-*luc2*-φC31-Neo plasmid was co-transfected with the φC31 integrase expression plasmid into HepG2 cells harboring the multi-integrase mouse artificial chromosome (MI-MAC) vector[7, 8] (a kind gift from Dr. M. Oshimura and Dr. Y. Kazuki of Tottori University) and sub-cultured for selection with 1 mg/ml G418 (Nacalai Tesque). The integration of expression plasmid into the corresponding sites on the MI-MAC vector was confirmed by a genomic polymerase chain reaction.

To measure the BL spectra of Fluc with D-LH_2 analogues in HepG2 cell lines (**2a-2c**), the stable HepG2 cells harboring the *luc2* gene with CAG promoter were grown in DMEM medium supplemented with 10% FBS, 1x minimum essential medium non-essential amino acids (Gibco), 1 mM sodium pyruvate (Gibco) and 25 mM HEPES-NaOH (pH 7.0), 5% CO_2 at 37 °C. The cells (1×10^6) were then seeded into a 35 mm Petri dish and maintained the growth for overnight. After that, the culture medium was discarded, and a solution of 100 µM of each D-LH_2 analogue (**2a-2c**) in DMEM medium (without phenol red) supplemented with 10% FBS and 25 mM HEPES-NaOH (pH 7.0) was added into the 35 mm Petri dish contained HepG2 cells. The BL signals of living HepG2 cells with **2a**-**2c** were monitored by a luminometer (ATTO, Kronos Dio, AB-2550 model). After the cells reached the highest BL intensity, the dish was recorded with a highly sensitive CCD spectrophotometer (ATTO, Lumi Fluoro Capture, AB-1850S) to measure the BL spectra of D-LH_2 analogues (**2a**-**2c**) in living HepG2 expressing Fluc. The number of HepG2 cell lines seeded into a 96-well plate was varied when testing with 100 µM of each D-LH_2 analogues (**2a**-**2c**) to determine the ability of **2a**-**2c** BL signal in live cells. The BL intensity of **2a**-**2c** in live HepG2 cells were monitored by a luminometer (ATTO, Kronos HT, WSL-1563) with F0 (no filter) and F2 (red filter, >620 nm long-pass filter, R62, Hoya) for 30 min. The BL intensity at the time of 30 min was collected and used for determining the lower limit of HepG2 cells needed for detection.

Transient transfection of luciferase reporter plasmids was prepared by reverse transfection techniques. The reverse transfection was performed using Lipofectamine 3000 (Thermo Fisher Scientific) according to the manufacturer's instructions. For each well of a 96-well plate, 200 ng of expression plasmids (pCAG-*luc2*, pCAG-*slr*, and pCAG-*pmluc*-N230S) were added in 25 µL of Opti-MEM (Gibco). Separately, 0.4 µL of Lipofectamine 3000 was added in 25 µL of Opti-MEM and incubated for 5 minutes at room temperature. The diluted

plasmids and Lipofectamine 3000 were then combined and incubated for 30 minutes to allow formation of DNA and lipid complexes. The DNA-lipid complexes were added directly to each well of the empty plate. 1.5×10^5 cells (HepG2 and HeLa cells) were then suspended in 150 μL of DMEM supplemented with 10% FBS were added directly into the transfection mixture in each well. The plate was gently agitated to ensure even distribution and incubated at 37°C in a humidified 5% CO_2 incubator. The reporter cells obtained at this stage were ready for further investigation of their bioluminescence with D-LH_2 analogues.

Real-time BL measurement was carried out in live HepG2 cells harboring *luc2* gene with D-LH_2 analogues (**2a-2c**). 15,000 HepG2 cells were seeded in a 96-well plate and then DMEM culture medium (without phenol red) supplemented with 100 μM of each D-LH_2 analogue (**2a-2c**), 10% FBS and 25 mM HEPES-NaOH (pH 7.0) was added and the BL signals were monitored with F0 (no filter) and F2 (red filter, >620 nm long-pass filter) by a luminometer (ATTO, Kronos HT, WSL-1563) and while maintaining the cells with 5% CO_2 at 37 °C.

1.7 Measurement of K_d values for firefly luciferase and luciferin binding

The bindings of firefly luciferase (Fluc) and D-LH_2 analogues were measured by MicroCal PEAQ-ITC technique (Malvern Panalytical). A solution of purified Fluc was dialyzed in 100 mM HEPES pH 8.0 at 4 °C for 16 hours. Then, the buffer outside the dialyzed enzyme was used to prepare a stock solution of each D-LH_2 analogue. To measure K_d values of Fluc and each D-LH_2 analogue complexes, 50 μM of D-LH_2 analogue solutions (**2a**-**2e**) were loaded into a sample cell and the Milli-Q Type I ultrapure water was added in a reference cell. A solution of 1.5 mM Fluc (2.0 μl) was continuously titrated into the sample cell (19 injections) until the ligand binding reached an equilibrium at 25 °C. The Microcal PEAQ-ITC analysis software was used to calculate a K_d value using the one-site binding model. We pre-incubated Fluc (1.5 mM) and ATP analogue (ApCPP (Adenosine-5'-[(α,β)-methyleno]triphosphate)) (1 mM) to form the Fluc-ApCCP complex to investigate effects of this preformed complex on the binding of luciferins to Fluc. A mixture of 1 mM ApCPP and 50 mM D-LH_2 analogue was loaded into a sample cell. The solution of 1.5 mM Fluc and 1 mM ApCPP was filled in a titration syringe and continuously titrated into the sample cell (2 μl in each injection for 19 injections). Heat changes from the binding were measured at 25 °C. The K_d values were calculated by the Microcal PEAQ-ITC analysis software.

1.8 Molecular docking and molecular dynamics (MD) simulations of luciferase and luciferin complexes

The crystal structure of Fluc (PDB 4G36)[9] was used as an initial structure for molecular docking. Complexes of Fluc and luciferins (D-LH_2 (**2a**), 5'-MeLH_2 (**2b**), and 5',7'-DiMeLH_2 (**2c**)) were prepared using AutoDockTools[10, 11] with the protonation states of the amino acids being predicted by PROPKA[12]. The active site in Chain A was selected as the center for a cubic docking box designated with dimensions of 50 Å^3 on each side. Molecular docking was performed using AutoDock Vina[13].

The crystal structure of Fluc (PDB 4G36)[9] was used for molecular dynamics (MD) simulations. The complexes of Fluc and luciferins from docking described above were used as starting structures for MD simulations. Hydrogen atoms of amino acid residues were added by considering results from the PROPKA[12]. The atom types in the topology files were assigned based on the CHARMM36 parameter set[14]. The structures of Fluc and ludiferin ligands were solvated in a cubic box of TIP3P water extending at least 15 Å from each direction in the solute. A dimension of the solvated system was 114 x 79 x 90 Å. The MD simulations protocols used were according to previous work published[15-17] [18] using the NAMD program[19] with simulation protocols adapted from our previous work[18, 20]. The simulations were started by minimizing hydrogen atom positions for 3,000 steps followed by water minimization for 6,000 steps. The system water was heated to 300 K for 5 ps and then was equilibrated for 15 ps. The whole system was minimized for 10,000 steps and heated to 300 K for 20 ps. After that, the whole system was equilibrated for 180 ps followed by a production stage for 40 ns. To investigate temperature effects on the enzyme stability, MD simulations were carried out at 300 - 360 K. Root mean square distances (RMSD) and binding of luciferins in Fluc at various temperatures were monitored, shown and discussed in the results section.

2. Supplementary Information

2.1 Identification of firefly luciferin (D-LH$_2$) and analogues

To synthesize the D-LH$_2$ (**2a**) and D-LH$_2$ analogues for further studies, the one-pot condensation reactions were performed (as described in section 1.1) and the products were identified by HPLC/ESI-QTOF-MS (positive mode) as shown in Table S1.

Supplementary Table S1. Identification of firefly luciferin (D-LH$_2$) and analogues analyzed by HPLC/ESI-QTOF-MS

Entry	Substrate	Product	Measured *m/z*	Ion formula	Theoretical *m/z*	Eror [mDa]	Adduct
1	*p*-benzoquinone (**1a**)	D-LH$_2$ (**2a**)	281.0078	$C_{11}H_9N_2O_3S_2$	281.0049	-2.9	M+H
2	*p*-toluquinone (**1b**)	5′-methyl-D-luciferin (5′-MeLH$_2$, **2b**)	295.0208	$C_{11}H_{10}N_2O_3S_2$	295.0206	-0.2	M+H
3	2,6-dimethyl-*p*-benzoquinone (**1c**)	5′,7′-dimethyl-D-luciferin (5′,7′-DiMeLH$_2$, **2c**)	309.0377	$C_{13}H_{12}N_2O_3S_2$	309.0362	-1.3	M+H
4	2,6-dimethoxy-*p*-benzoquinone (**1d**)	5′,7′-dimethoxy-D-luciferin (5′,7′-DiOMeLH$_2$, **2d**)	341.0265	$C_{13}H_{12}N_2O5S_2$	341.0260	-0.4	M+H
5	*p*-xyloquinone (**1e**)	4′,7′-dimethyl-D-luciferin (4′,7′-DiMeLH$_2$, **2e**)	309.0364	$C_{13}H_{12}N_2O_3S_2$	309.0362	-0.2	M+H
6	2-methyl-*p*-naphthoquinone (**1f**)	7′-methyl naphtho-D-luciferin (7′-MeNpLH$_2$, **2f**)	345.0355	$C_{16}H_{12}N_2O_3S_2$	345.0362	0.7	M+H

2.2 Effects of buffer type on D-LH$_2$ synthesis

The D-LH$_2$ (**2a**) synthesis reactions containing 1 mM BQ (**1a**), 2 mM D-Cys in various buffers and pHs, *i.e.,* 100 mM of HEPES-NaOH (pH 8.0), sodium phosphate (NaPi-NaOH) (pH 7.5 and 8.0), Tris-HCl (pH 8.0), ammonium acetate (NH_4OAc) (pH 8.0), and ammonium bicarbonate (NH_4HCO_3) (pH 8.0), were carried out at room temperature. Samples were prepared and analyzed by HPLC/ESI-QTOF-MS (positive mode). The results indicate that NaPi-NaOH, Tris-HCl, NH_4OAc and NH_4HCO_3 gave lower yields of **2a** than the reactions in HEPES-NaOH (pH 8.0) as shown in Fig. S1a. We then varied the concentration of HEPES buffer to minimize the cost. The results in Fig. S1b showed that 100 mM HEPES gave the best yield of **2a**, possibly due to higher ionic strengths than other conditions.

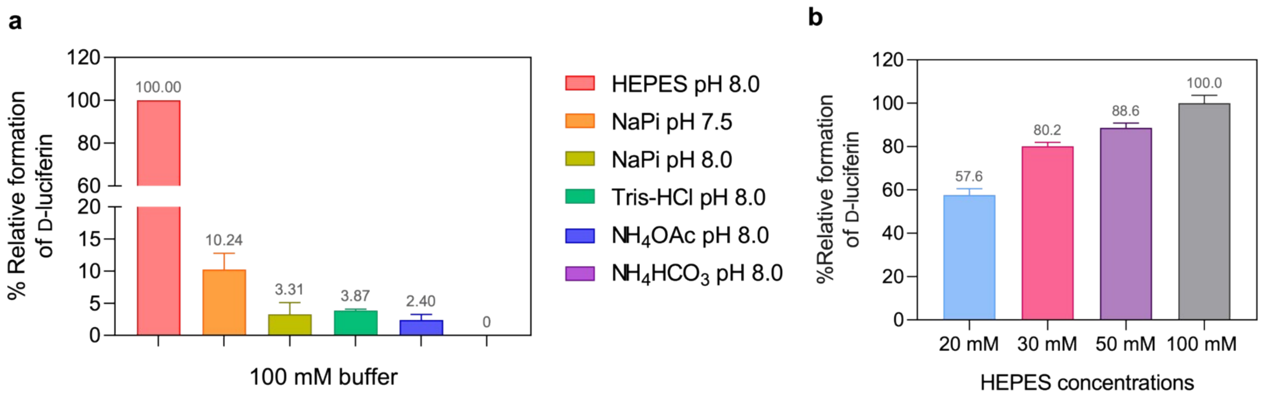

Supplementary Fig. S1 Effects of buffer on yield of D-LH_2. a) 100 mM buffers with a pH range of 7.5 to 8.0 including HEPES, NaPi, Tris-HCl, and NH_4OAc were tested. The results indicate that 100 mM HEPES pH 8.0 gave the best yield of D-LH_2 (**2a**) formation. b) Lower the concentration of HEPES lowered the yield of **2a**.

2.3 Effects of co-solvent on D-LH_2 formation

To synthesize D-LH_2 (**2a**) and its analogues with a green chemistry method, the reactions were carried out in different co-solvent to boost the yield of **2a**. A solution (5 ml) containing 1 mM **1a** and 2 mM D-Cys was mixed in 100 mM HEPES-NaOH (pH 8.0). Co-solvents including MeOH, EtOH, ACN, (+)-limonene, and *p*-cymene were then added to explore the effects on yield enhancement. The reaction without addition of co-solvent was used as a benchmark for comparing the yield with co-solvent addition. The results indicate that the 20–30% (v/v) MeOH addition can increase **2a** yield by about 2.8-fold. The addition of EtOH produced a comparable outcome, increasing the yield of **2a** production by nearly three times with 50% (v/v) EtOH. However, addition of higher concentration (> 20% v/v) of EtOH caused D-Cys precipitation. Therefore, the addition of 20% (v/v) of MeOH is a suitable concentration to stabilize the **1a** substrate[21] and to improve the yield of **2a**.

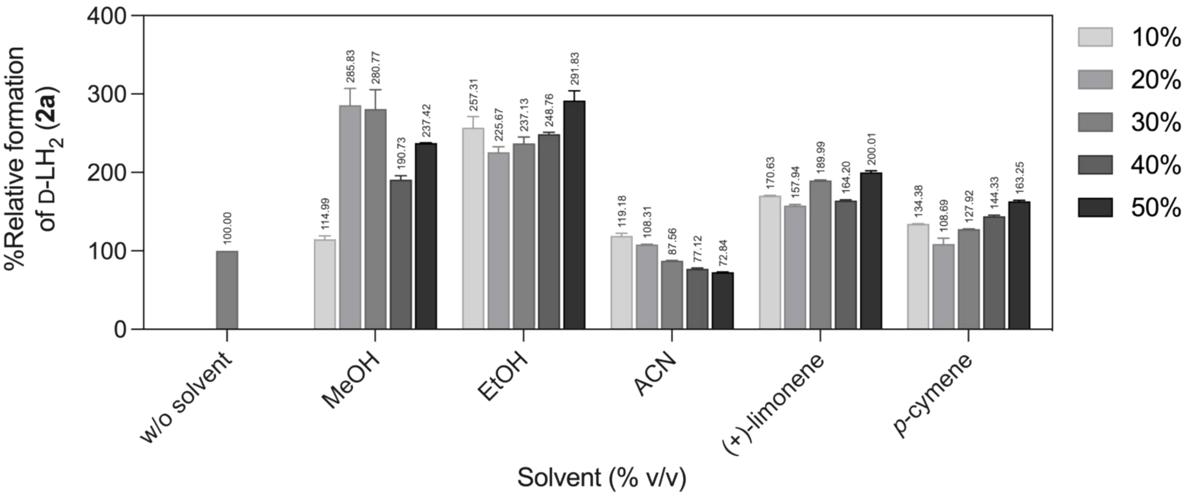

Supplementary Fig. S2 Effects of solvent on D-LH_2 (**2a**) formation. Different co-solvents including MeOH, EtOH, ACN, (+)-limonene, and *p*-cymene at various concentrations were explored. The addition of ACN cannot enhance the formation of **2a** when compared to the benchmark reaction (without addition of solvent). The bio-based solvents including (+)-limonene and *p*-cymene slightly enhanced the production yield of **2a** about 1.2-2.0-fold, while 20 % (v/v) MeOH and 50 % (v/v) EtOH promoted the **2a** formation better than the benchmark about 2.85- and 2.98-fold, respectively.

2.4 New firefly luciferins and their structural elucidation

A semi large-scale one-pot reaction of D-LH_2 analogues (**2c-2f**) synthesis was carried out in a 100-ml reaction. The compounds were purified as described in Section 1.4. The purified D-LH_2 analogues (**2c-2f**) were identified by reverse phase thin layer chromatography (RP-TLC) and further characterized by NMR spectroscopy. The assigned NMR peaks of D-LH_2 (**2a**) and analogues (**2c-2f**) based on ^{1}H- and ^{13}C-NMR spectra are shown in Tables S2 and S3, respectively.

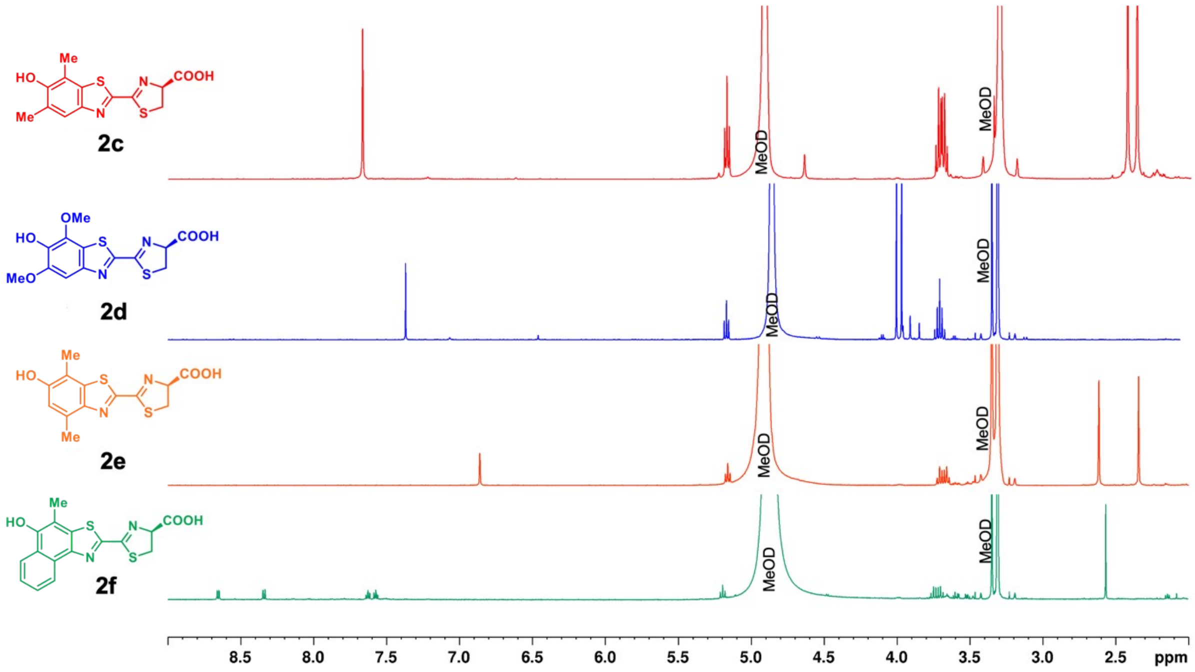

Supplementary Fig. S3 ^{1}H NMR spectra of luciferin analogues. Spectra shown are those of 5′,7′-dimethyl luciferin (5′,7′-DiMeLH$_2$, **2c**, red), 5′,7′-dimethoxy luciferin (5′,7′-DiMeOLH$_2$, **2d**, blue), 4′,7′-dimethyl luciferin (4′,7′-DiMeLH$_2$, **2e**, orange), and 7′-methylnaphthol luciferin (7′-MeNpLH$_2$, **2f**, green).

Supplementary Table S2. ^{1}H NMR spectroscopic data of D-LH$_2$ analogues from a one-pot non-enzymatic reactions.

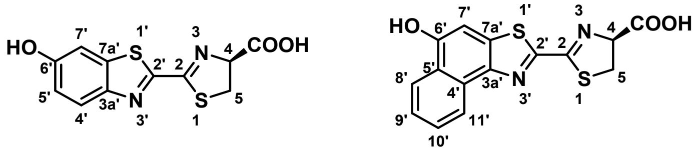

D-luciferin (D-LH$_2$) **Naphtho-luciferin (NpLH$_2$)**

Position	5′,7′-DiMeLH$_2$ (**2c**)	5′,7′-DiOMeLH$_2$ (**2d**)	4′,7′-DiMeLH$_2$ (**2e**)	7′-MeNpLH$_2$ (**2f**)
H-4	5.18 (t, *J* = 9.4 Hz, 1H)	5.17 (t, *J* = 9.4 Hz, 1H)	5.16 (t, *J* = 9.5 Hz, 1H)	5.19 (t, *J* = 9.3 Hz, 1H)
H$_a$-5/H$_b$-5	3.71(m, 2H)	3.71 (m, 2H)	3.68 (m, 2H)	3.72 (m, 2H)
H-4′	7.67 (s, 1H)	7.37 (s, 1H)	-	-
H-5′	-	-	6.86 (s, 1H)	-
H-7′	-	-	-	-
H-8′	-	-	-	8.34 (d, *J* = 8.1 Hz, 1H)
H-9′	-	-		7.57 (t, *J* = 7.8 Hz, 1H)
H-10′	-	-	-	7.62 (t, *J* = 7.6 Hz, 1H)
H-11′	-	-	-	8.65 (d, *J* = 7.9 Hz, 1H)
4′-Me	-	-	2.34 (s, 3H)	-
5′-Me	2.37 (s, 3H)	3.97 (s, 3H)	-	-
7′-Me	2.43 (s, 3H)	4.00 (s, 3H)	2.61 (s, 3H)	2.57 (s, 3H)

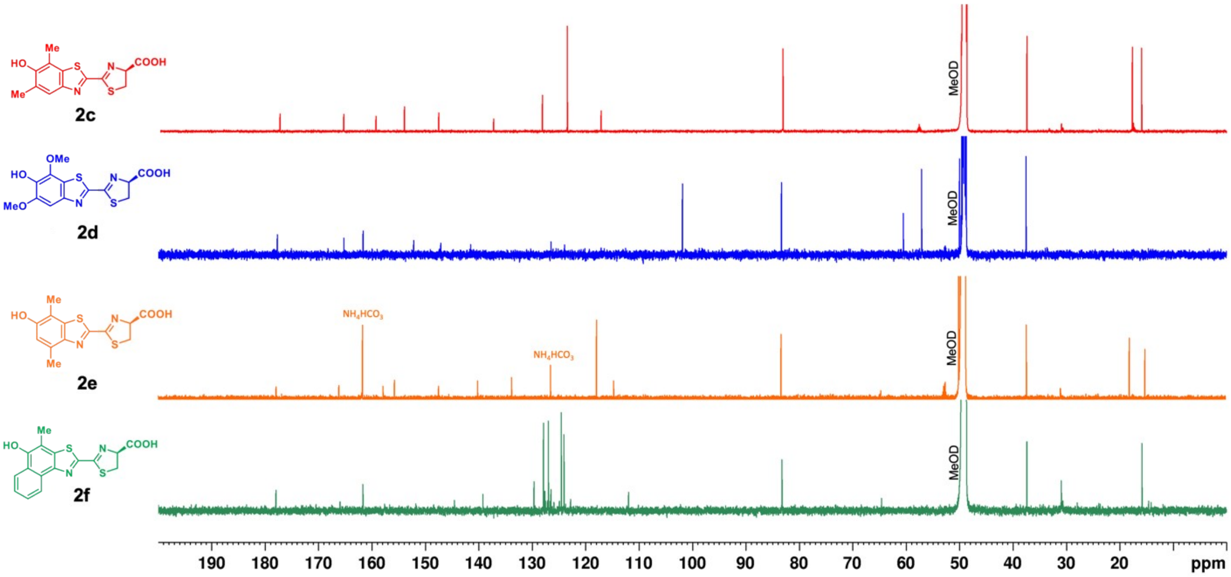

Supplementary Fig. S4 ^{13}C NMR spectra of luciferin analogues. Spectra shown are those of 5′,7′-dimethyl luciferin (5′,7′-DiMeLH$_2$, **2c**, red), 5′,7′-dimethoxy luciferin (5′,7′-DiMeOLH$_{2,}$ **2d**, blue), 4′,7′-dimethyl luciferin (4′,7′-DiMeLH$_2$, **2e**, orange), and 7′-methylnaphthol luciferin (7′-MeNpLH$_2$, **2f**, green). Because ammonium bicarbonate (NH_4HCO_3) was used as in the elution buffer during purification process, the compound was found in the ^{13}C NMR spectrum of **2e**.

Supplementary Table S3. ^{13}C NMR spectroscopic data of D-LH$_2$ analogues from a one-pot non-enzymatic reactions.

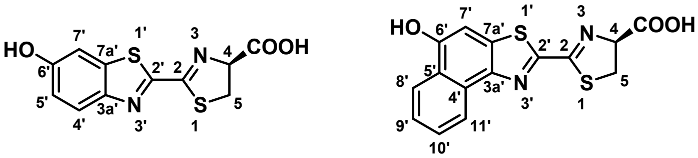

D-luciferin (D-LH$_2$) **Naphtho-luciferin (NpLH$_2$)**

Position	5′,7′-DiMeLH$_2$ (**2c**)	5′,7′-DiOMeLH$_2$ (**2d**)	4′,7′-DiMeLH$_2$ (**2e**)	7′-MeNpLH$_2$ (**2f**)
COOH	177.5	177.7	177.9	177.9
2	165.6	165.2	166.1	165.9
4	83.2	83.3	83.3	83.2
5	37.4	37.5	37.3	37.4
2′	159.5	161.7	157.8	161.7
3a′	147.8	150.2	147.4	144.5
4′	123.6	101.8	133.7	126.5
5′	128.3	147.1	117.8	127.6
6′	154.2	126.5	155.7	129.7
7′	117.3	141.6	114.6	112.0
7a′	137.5	124.0	140.1	139.2
8′	-	-	-	127.9
9′	-	-	-	124.0
10′	-	-	-	124.5
11′	-	-	-	127.1
4′-Me	-	-	18.0	-
5′-Me	17.7	60.5	-	-
7′-Me	15.9	57.1	15.1	15.8

2.5 Effects of oxygen on D-LH$_2$ formation

Normally, a one-pot non-enzymatic reaction of D-LH$_2$ (**2a**) synthesis was carried out under ambient atmospheric condition (20 % O_2). To further improve the yield of **2a**, the effects of O_2 were investigated. The conditions were set up similar to those described in Section 1.1) with various concentrations of O_2, including 20%, 10% and <0.0005% O_2 (anaerobic condition), respectively. Samples were collected every hour for up to 8 hours and then again at 23 and 24 hours. The samples were prepared and analyzed using HPLC-QQQ-MS (positive mode). Results in Fig. S3a showed that the reaction under anaerobic conditions gave the highest amount of **2a**, which was 1.8-fold greater than the benchmark condition (20% O_2, ambient air). We also investigated the rate of product formation, and the results are shown in Fig. S3b-d and Table S4. For the reaction under 20% O_2, the **2a** formation rate was 4.38 μM/h. For the formation rate of **2a** under 10% O_2 and anaerobic conditions, the rate were 3.45 and 3.26 μM/h, respectively. The results showed that O_2 concentration can increase the rate of **2a** formation as well as the instability of substrates (**1a** and D-Cys), resulting in an overall low yield of **2a** (Table S4)

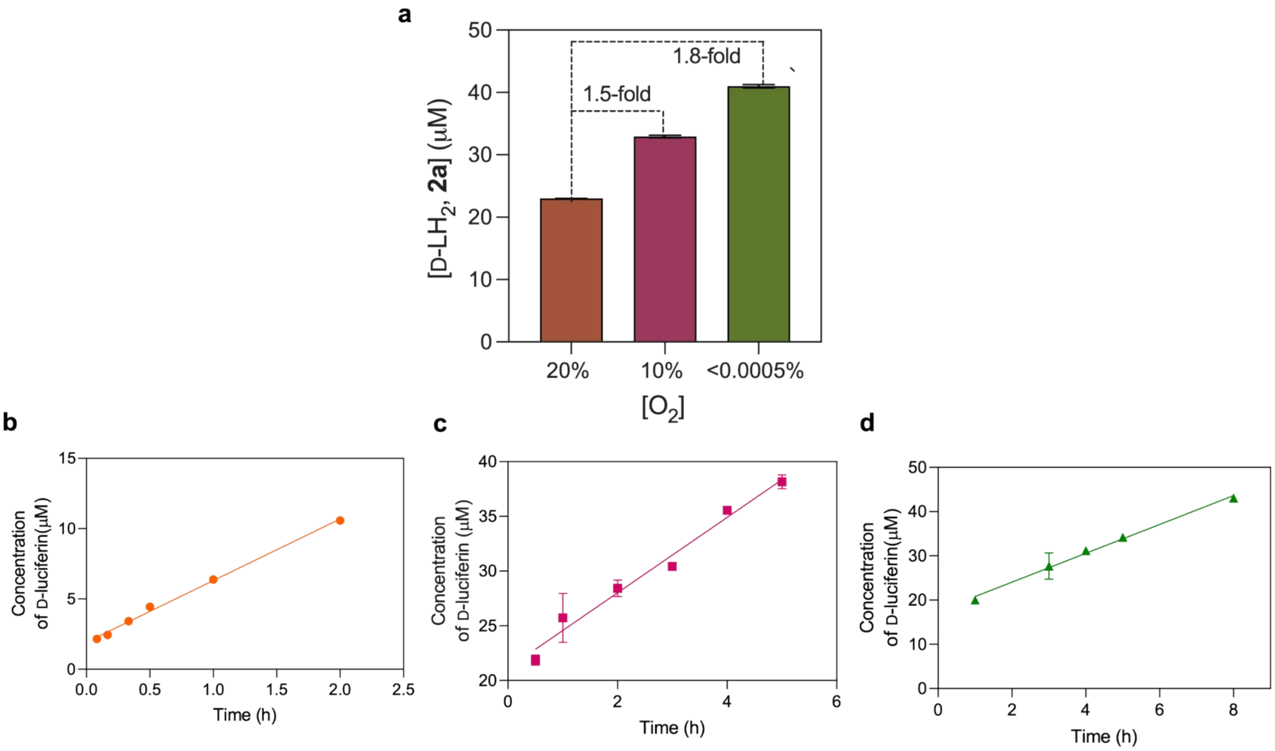

Supplementary Fig. S5 Effects of oxygen (O_2) concentrations on D-LH$_2$ (**2a**) formation. **a**) Comparison of **2a** formation yield under various concentrations of O_2. b-d) Rates of **2a** formation were determined under various O_2 concentrations from 20% (ambient), 10% and <0.0005% (anaerobic condition), respectively.

Supplementary Table S4. Effects of O_2 concentrations on D-LH$_2$ (**2a**) formation and rate of **2a** formation under different O_2 concentrations.

Entry	% O_2 concentration	Rate (μM/h)	Concentration of luciferin at 24h (μM)	% Yield at 24h	Fold-change
1	20% O_2	4.38 ± 0.02	23.00 ± 0.01	2.30 ± 0.00	-
2	10% O_2	3.95 ± 0.86	32.90 ± 0.21	3.30 ± 0.02	1.5
3	<0.0005% O_2	3.26 ± 0.14	41.00 ± 0.25	4.10 ± 0.03	1.8

2.6 Spectra of firefly luciferins

To determine the spectroscopic characteristics of D-LH_2 analogues (**2a-2f**), absorption, fluorescence and BL spectra of the purified D-LH_2 analogues (**2a-2f**) in 100 mM HEPES-NaOH (pH 8.0) were recorded. The absorption spectra of **2a-2f** were recorded by a double-beam spectrophotometer (Agilent technologies, Cary 100 UV Vis, Cary Series UV-Vis Spectrophotometer). The fluorescence spectra of **2a-2f** were recorded by a microplate reader (Thermo Scientific, Varioskan, LUX multimode) in fluorescence mode. The reactions of D-LH_2 analogues (**2a-2f**) and luciferases were carried out and their bioluminescence spectra were recorded. Solutions of **2a-2f** in 100 mM HEPES-NaOH (pH 8.0) were mixed in 100 mM HEPES-NaOH pH 8.0 containing 10 μM luciferase (Fluc, SLR, Eluc, Pmluc-WT, Pmluc-N230S), 3 mM ATP, and 6 mM $MgCl_2$ and the BL spectra were immediately measured by a microplate reader (Thermo Scientific, Varioskan, LUX multimode) using BL mode. The results indicate that substitutions of a methyl (CH_3) group into the benzothiazole moiety of luciferins **2b**, **2c**, and **2e** do not significantly alter the maximum wavelength (λ) of absorption and fluorescence spectra of the natural D-LH_2 (**2a**). However, the substitution with methoxy (OCH_3) and aromatic groups in **2d** and **2f** significantly change the absorption and fluorescence spectra as shown in Table S5. In addition, the substitution of methyl, methoxy, and aromatic groups (**2b-2f**) at the benzothiazole ring of luciferins provide the longer wavelength of BL spectra than 600 nm. The longest red-shifted BL signals could be obtained from the reactions of 5',7'-DiOMeLH$_2$ (**2d**) with SLR at 663 nm and with Pmluc-N230S at 646 nm (Table S5). Because of their red-shifted light emission features, the BL measurement of D-LH_2 analogues suggests that these compounds could potentially be used in future applications for bioimaging purposes.

Supplementary Table S5. Absorption, fluorescence, and bioluminescence characteristics of D-LH_2 and D-LH_2 analogues

Entry	D-LH_2 analogues	% Purity[a]	% yield[b]	Structure	Absorption (λ), nm	Fluorescence (^{em}λ), nm	Bioluminescence (λ), nm				
							Fluc	SLR	Eluc	Pmluc-WT	Pmluc-N230S
1	**2a**	95.0	5.0 ± 0.1[c]<br>0.30 ± 0.24 [22]<br>46.0 [1]<br>42.0 [23]<br>50[d]	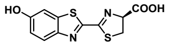	327	543	560	625	537	554	614
2	**2b**	95.0	15.0 ± 0.1[c]<br>By product [24]	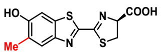	323	523	605	626	560	608	608
3	**2c**	97.0	4.3 ± 0.5[c]<br>88.0 [25]<br>66[d]	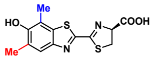	334	560	628	634	573	631	628
4	**2d**	94.0	2.0 ± 0.3[c]	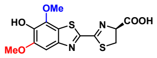	412	620	625	663	596	582	646
5	**2e**	95.0	19.2 ± 0.1[c]<br>64.0 [26]	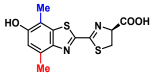	330	560	636	644	583	637	631
6	**2f**	91.0	6.7 ± 0.1[c]	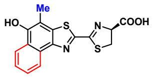	437	580	648	634	557	ND[e]	ND[e]

[a] NMR spectroscopy was used to determine the purity of D-LH_2 analogues. [b] The overall yield from conventional chemical synthesis route. [c] The isolated yield from the synthesis and purification method reported in this work. [d] The overall yield obtained from using the recently reported protocol[1] carried out in this work. [e] ND denotes "not detectable".

2.7 Steady-state kinetics of the reactions of luciferases and D-LH_2 analogues

Kinetic parameters of luciferases and D-LH_2 analogues reactions were determined to compare with those of natural D-LH_2. The 150 μl reactions were set up in 100 mM HEPES-NaOH (pH 8.0) and measured by a luminometer (Luminescencer-Octa, AB-2270, ATTO). The premixed luciferases/D-LH_2 analogues solution of 25 μl was mixed with 125 μl of the 3 mM ATP, 6 mM $MgCl_2$ solution to initiate the BL reading by a luminometer for 300 seconds. To determine the BL intensities (BLI) of the light produced by these D-LH_2 analogues, the RLUs or counts/s (CPS) were measured for 300 seconds. Relative k_{cat} values (quantum yield × k_{cat}) were calculated by dividing BLI values (BLI= quantum yield × k_{cat} × [E]) with the concentration of luciferases (Fluc, SLR, Eluc, Pmluc-WT, and Pmluc-N230S). BLI at various concentrations of D-LH_2 analogues were used for analyzing kinetic parameters using the Michaelis-Menten equation to obtain the relative

k_{cat}, V_{max}, and K_m values (Table S6). k_{cat} values were calculated using Equation (1) and the values were used to evaluate the efficiency of D-LH_2 analogues in the luciferase reactions (see the Main text Fig. 5a).

$$k_{cat} = V_{max} / [E]_t \qquad \text{Equation (1)}$$

Where;
k_{cat} is a catalytic turnover
V_{max} is a maximum velocity
$[E]_t$ is a total concentration of enzyme in the reaction

Supplementary Table S6. Kinetic parameters of the reactions of luciferases with D-LH_2 analogues (**2a-2f**).

Entry	D-LH_2 analogues	Luciferase	$^{app}K_m$, μM	V_{max}, CPS	k_{cat}, CPS/nM	Relative k_{cat}[a]	Substrate inhibition (μM)
1	**2a**	Fluc	3.3	2.1×10^8	2.8×10^6	100.0 [14]	> 10.0
	**2b**		3.1	4.9×10^8	6.5×10^6	232.1 [14]	> 10.0
	**2c**		2.5	14.4×10^7	1.9×10^6	67.8	> 12.5
	**2d**		0.2	2.8×10^6	3.7×10^4	1.3	> 0.8
	**2e**		7.1	2.2×10^6	2.9×10^4	1.0	> 30.0
	**2f**		2.7	9.5×10^4	2.5×10^3	0.1	> 100.0
2	**2a**	SLR	0.5	6.7×10^7	8.9×10^5	100.0	> 4.0
	**2b**		0.7	10.3×10^7	1.4×10^6	157.3	> 14.0
	**2c**		0.5	9.4×10^7	1.2×10^6	134.8	> 2.1
	**2d**		5.0	5.0×10^5	6.7×10^3	0.8	> 16.6
	**2e**		9.0	3.7×10^6	4.9×10^4	5.5	> 33.3
	**2f**		11.6	3.9×10^5	1.0×10^4	1.1	> 100.0
3	**2a**	Eluc	0.1	10.3×10^8	1.4×10^7	100.0	> 1.0
	**2b**		0.7	8.8×10^7	1.2×10^6	8.6	> 8.0
	**2c**		3.5	3.5×10^6	4.7×10^4	0.3	> 12.5
	**2d**		34.7	8.8×10^5	1.2×10^4	0.1	ND[b]
	**2e**		19.1	1.9×10^6	2.5×10^4	0.2	> 66.6
	**2f**		24.7	1.4×10^4	3.6×10^2	0.0	> 200.0
4	**2a**	Pmluc-WT	0.1	7.6×10^8	1.0×10^7	100.0	> 4.0
	**2b**		0.4	8.4×10^8	1.1×10^7	110.0	> 2.0
	**2c**		1.8	5.8×10^7	7.7×10^5	7.7	> 8.3
	**2d**		0.2	1.2×10^5	1.6×10^3	~0.02	> 0.8
	**2e**		10.2	8.8×10^6	1.2×10^5	1.2	> 33.3
	**2f**		ND[b]	ND[b]	ND[b]	ND[b]	ND[b]
5	**2a**	Pmluc-N230S	0.6	2.0×10^8	2.7×10^6	100.0	> 4.0
	**2b**		1.8	10.4×10^8	1.4×10^7	518.5	> 4.0
	**2c**		2.6	10.0×10^7	1.3×10^6	48.1	ND[b]
	**2d**		13.3	4.4×10^5	5.9×10^3	0.2	> 41.6
	**2e**		6.6	1.3×10^7	1.7×10^5	6.3	> 33.3
	**2f**		10.0	8.6×10^3	2.3×10^2	~0.01	> 100.0

[a] is relative to **2a** compound, [b] ND denotes "not detectable" at the concentration range tested (0-300 μM)

2.8 Investigation of bioluminescence kinetics using stopped-flow luminometry

To explore the kinetics of BL emission for firefly luciferase (*photinus pyralis* luciferase, Fluc) with D-LH_2 analogues (**2a**-**2d**), kinetics of their reactions were investigated using a stopped-flow spectrophotometer (employing a light-off mode for detecting the luminescence). One syringe was filled with a solution containing 50 μM Fluc, 3 mM ATP, and 6 mM $MgCl_2$ in 100 mM HEPES-NaOH (pH 8.0) while another syringe was filled with a solution of 10 μM D-LH_2 analogues (**2a**-**2d**) in 100 mM HEPES-NaOH (pH 8.0). Solutions in both syringes were mixed in a stopped-flow device and light emission characteristics were measured at 25 °C. Each experiment was carried out at least in triplicate. Fig. S4 and Table S7 showed the average of kinetic traces and their corresponding observed rate constants (k_{obs}), respectively.

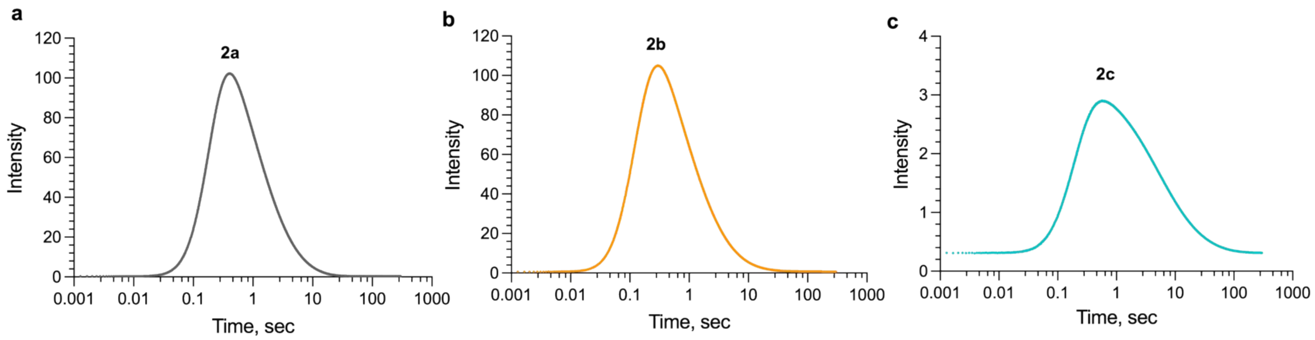

Supplementary Fig. S6 Kinetics of light formation and decay of Fluc reactions with D-LH_2 analogues (**2a**-**2d**) carried out by a stopped-flow spectrophotometer. A single turnover reaction of Fluc with a) natural D-LH_2 (**2a**), b) 5'-MeLH_2 (**2b**), and c) 5',7'-DiMeLH_2 (**2c**).

Supplementary Table. S7. Summary of rate constants of light formation and decay of Fluc reactions with D-LH_2 analogues

Entry	D-LH_2 analogues	Light formation (k_{obs}, s^{-1})	Light decay (k_{obs}, s^{-1})	
			Faster rate	Slower rate
1	**2a**	6.64 ± 0.05	1.57 ± 0.02	0.29 ± 0.00
2	**2b**	9.63 ± 0.04	1.55 ± 0.15	0.27 ± 0.00
3	**2c**	7.28 ± 0.02	0.27 ± 0.00	0.05 ± 0.00

2.9 Measurement of quantum yield values

Quantum yield (QY) of luciferase-luciferin pairs was measured and calculated as shown in Table S8. The red-shifted D-LH_2 analogue **2b** gave a greater QY value than that of natural D-LH_2 (**2a**) in the Fluc reaction, while **2b** provided similar QY values to that of **2a** in the reactions of SLR and Pmluc-N230S. Moreover, the **2c** compound provided reasonable QY values in the reactions of Fluc, SLR, and Pmluc-N230S. However, the **2d** compound gave low bioluminescence and their QY values could not be determined.

Supplementary Table S8. Summary of quantum yield (QY) values and related parameters.

Luciferase	Luciferin	λ_{max}, nm	Photon[a] (x 10^{11})	Quantum yield
Fluc	**2a**	560	2.68 x 10^{11}	0.44 ± 0.040
	**2b**	605	3.13 x 10^{11}	0.52 ± 0.010
	**2c**	628	1.23 x 10^{11}	0.20 ± 0.007
	**2d**	625	ND[b]	ND[b]
SLR	**2a**	625	1.09 x 10^{10}	0.16 ± 0.005
	**2b**	626	1.04 x 10^{10}	0.19 ± 0.005
	**2c**	634	9.70 x 10^{9}	0.16 ± 0.003
	**2d**	663	ND[b]	ND[b]
Pmluc-N230S	**2a**	614	9.50 x 10^{9}	0.16 ± 0.018
	**2b**	608	1.01 x 10^{10}	0.17 ± 0.034
	**2c**	628	8.79 x 10^{9}	0.15 ± 0.014
	**2d**	646	ND[b]	ND[b]

[a] is average of accumulated total number of photons emitted from the concentration range (6.02-60.2 x 10^{10}) of reacted D-LH_2 analogues (**2a**-**2c**).
[b] ND denotes "not determined".

2.10 Measurement of K_d and thermodynamics parameters by ITC

Isothermal Titration Calorimetry (ITC) was used for measuring K_d values and thermodynamics parameters of binding of firefly luciferase (Fluc) and D-LH_2 analogues (**2a**-**2e**). Results in Fig. S7 and Table S9 showed that all D-LH_2 analogues (**2a**-**2d**) except the **2e** compound can bind reasonably well to Fluc. This may be due to that the methyl substitution at position 4', and 7' of benzothiazole ring of **2e** may obstruct its interactions with Fluc; the K_d value of **2e** binding to Fluc thus could not be determined using ITC. Without the addition of ATP analogue, the **2b** compound revealed the highest binding affinity to Fluc than other compounds. However, the pre-formation of Fluc with ApCCP (ATP analogue) complex can facilitate the binding all D-LH_2 analogues as their the K_d values were lower under this condition (Table S9).

All ITC curves in Fig. S7 showed downward peaks which indicate the exothermic reactions. Therefore, the thermodynamic binding profiles of Fluc and D-LH_2 analogues (**2a**-**2d**) were enthalpic-driven interactions (Table S9). Thermodynamics parameters in Table S9 indicate that the binding of Fluc and D-LH_2 analogues at 25 °C are all thermodynamically favorable with negative values of ΔG for all bindings.

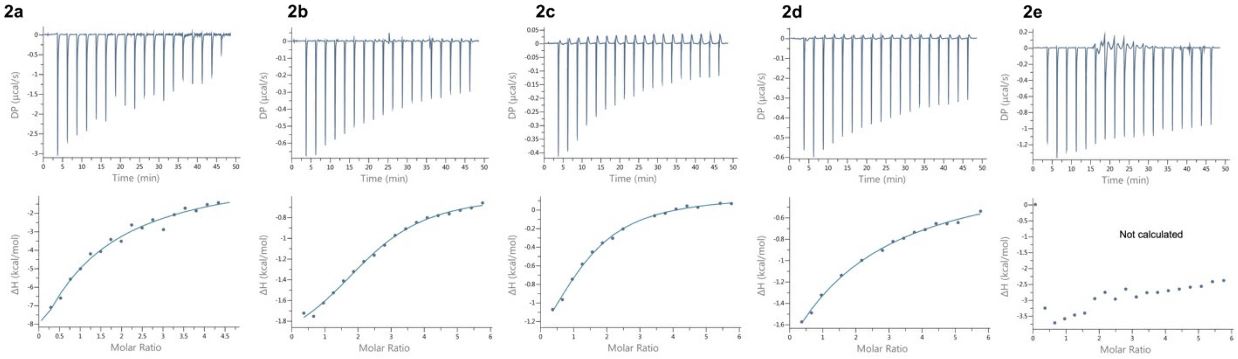

Supplementary Fig. S7. Isothermal Titration Calorimetry (ITC) analysis for binding of Fluc and D-LH_2 analogues (**2a**-**2e**).

Supplementary Table S9. Summary of ITC analysis for binding of Fluc and D-LH_2 analogues and thermodynamics parameters

Luciferase-Luciferin	K_d, μM	N site	ΔH, kcal/mol	ΔG, kcal/mol
Fluc-**2a**	21.1 ± 7.4	1.00 ± 0.28	-6.17 ± 2.10	-6.32 ± 0.20
Fluc-**2b**	13.4 ± 2.5	0.70 ± 0.18	-1.83 ± 1.55	-6.70 ± 0.11
Fluc-**2c**	35.1 ± 3.1	0.80 ± 0.10	-3.20 ± 0.21	-6.08 ± 0.05
Fluc-**2d**	30.4 ± 8.2	0.42 ± 0.05	-5.24 ± 2.05	-6.18 ± 0.17
Fluc-**2e**	NC[a]	NC[a]	NC[a]	NC[a]
Fluc-ApCCP-**2a**[b]	4.2 ± 0.1	0.32 ± 0.02	-7.65 ± 1.17	-7.00 ± 0.20
Fluc-ApCCP-**2b**[b]	4.00 ± 0.02	0.33 ± 0.10	-4.60 ± 0.37	-7.60 ± 0.42
Fluc-ApCCP-**2c**[b]	25.3 ± 1.1	3.34 ± 2.11	-4.85 ± 6.03	-6.01 ± 0.08
Fluc-ApCCP-**2e**[b]	864 ± 7	0.90 ± 0.44	-80.0 ± 0.00	-4.41 ± 0.40

[a] NC denotes "not calculated" by ITC because a K_d value for **2e** binding to Fluc was high.
[b] The K_d values measured by titration between the Fluc-ApCCP complex and D-LH_2 analogues. ApCPP (Adenosine-5'-[(α,β)-methyleno]triphosphate) is an ATP analogue which cannot react with luciferins.

2.11 Molecular docking and molecular dynamics simulations of the complexes of beetle luciferase and D-LH_2 analogues

To explore the binding mode of D-LH_2 analogues in the active site of luciferases, we performed molecular docking of **2a**-**2e** into Fluc using AutoDock vina. The binding energy shown in Figs. S8a-e revealed that all D-LH_2 analogues (**2a**-**2e**) bind to the active site of Fluc reasonably well, in agreement with the ITC results (see main text in Table 2 and Table S9).

2.11.1 Molecular docking of Fluc with 2a-2e

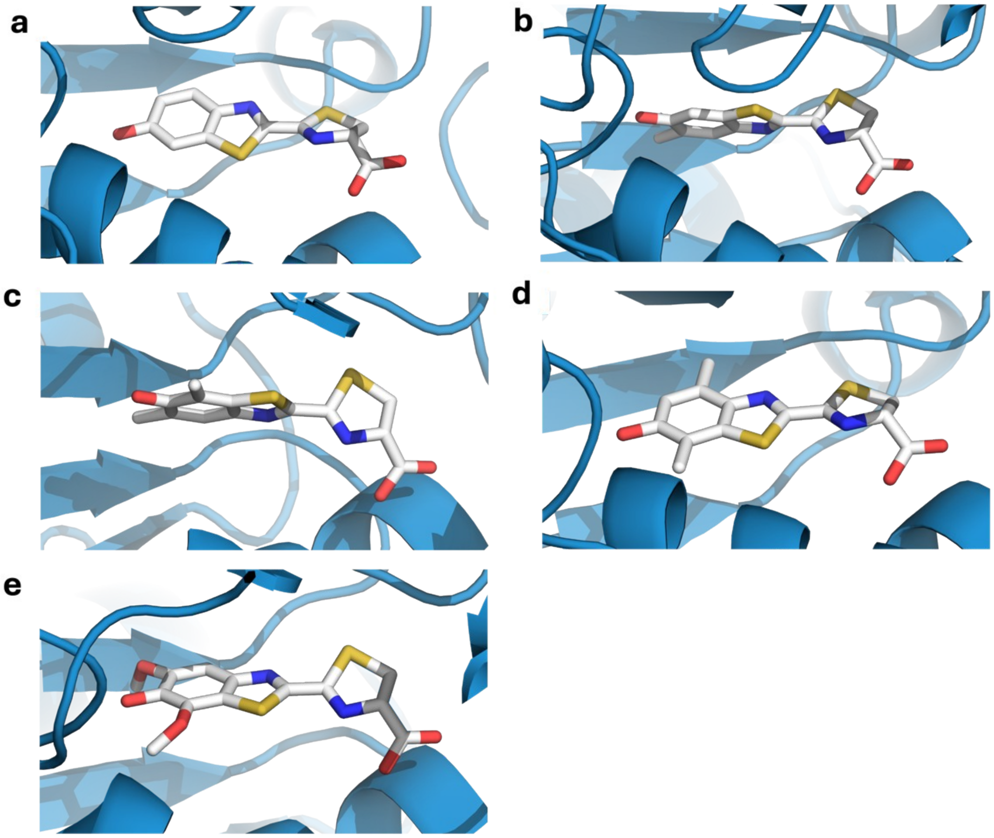

Supplementary Fig. S8. Binding of luciferins in Fluc modeled by molecular docking. a) D-LH_2 (**2a**). b) 5′-MeLH_2 (**2b**). c) 5′,7′-DiMeLH_2 (**2c**). d) 4′,7′-DiMeLH_2 (**2d**). e) 5′,7′ DiOMeLH_2 (**2e**).

2.11.2 Molecular dynamics (MD) simulations of Fluc with 2a-2e

The binding interactions of D-LH_2 (**2a**), 5′-MeLH_2 (**2b**) and 5′,7′-DiMeLH_2 (**2c**) with Fluc were further examined using MD simulations. The ligand-protein interactions analyzed by MD simulations at 300 K shown by LigPlot revealed that the hydroxyl group on the benzothiazole ring and the carboxylic group on the thiazoline ring of both **2a** and **2b** form hydrogen bonds with active site residues (Fig. S9 and S10). In addition to hydrogen bonding, these ligands interact with a greater number of active site residues through hydrophobic interactions compared to **2c** (Fig. S11), suggesting that **2a** and **2b** can bind more effectively to Fluc than does **2c**.

To further investigate the binding stability of the D-LH_2 analogues, MD simulations were performed across a range of temperatures (300–360 K). As shown in Figs. S12–15, the structure of Fluc bound to 5′-MeLH_2 (5ML, **2b**) and natural D-LH_2 (LUC, **2a**) remained stable across all temperatures tested, particularly at the elevated temperature of 360 K compared to 5′,7′-DiMeLH_2 (57D, **2c**). Structural analyses revealed that **2a** and **2b** remained bound within the Fluc active site from 300 to 360 K (Fig. S16 and S17), whereas **2c** maintained active site binding only between 300 and 340 K (Fig. S18). At 360 K, **2c** dissociated from the active site and relocated near the protein surface (Fig. S18a and S18e). These findings suggest that **2a** and **2b** exhibit stronger binding to Fluc compared to **2c** which correlate with the results from the protein-ligand interaction analysis described above.

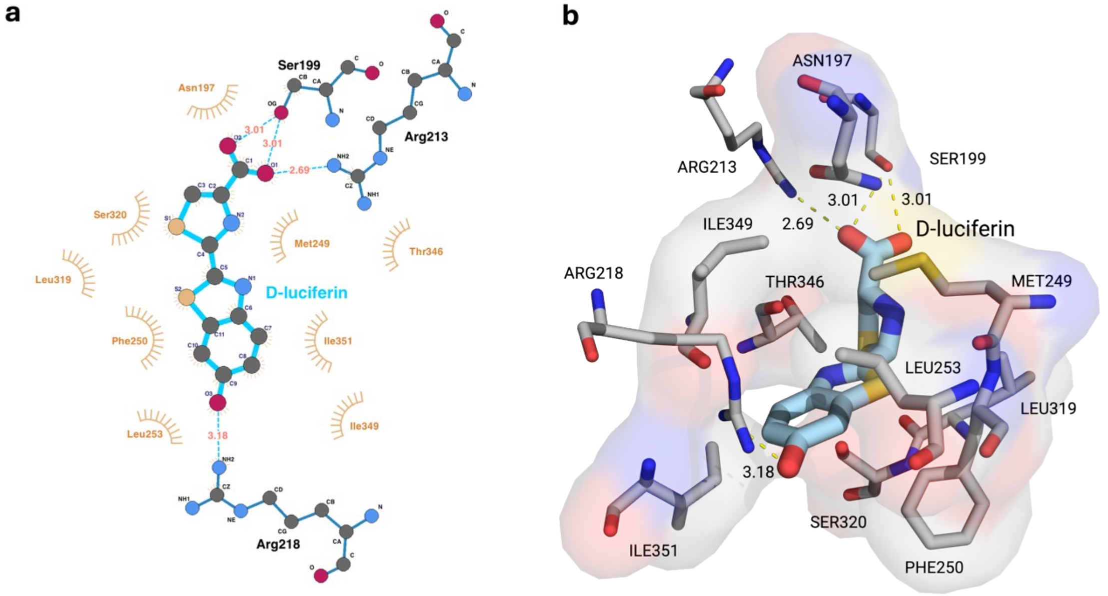

Supplementary Fig. S9. Interactions of active site residues with D-luciferin (D-LH_2, **2a**). a) The interactions of active site residues with **2a** shown by LigPlot. Hydrogen bonds are shown as blue dashed lines, while the spoked arcs represent protein residues making nonbonded contacts with the ligand. b) 3D structure of active site residues interacting with **2a** based on the LigPlot analysis. The analysis used MD results of temperature at 300K and 40 ns.

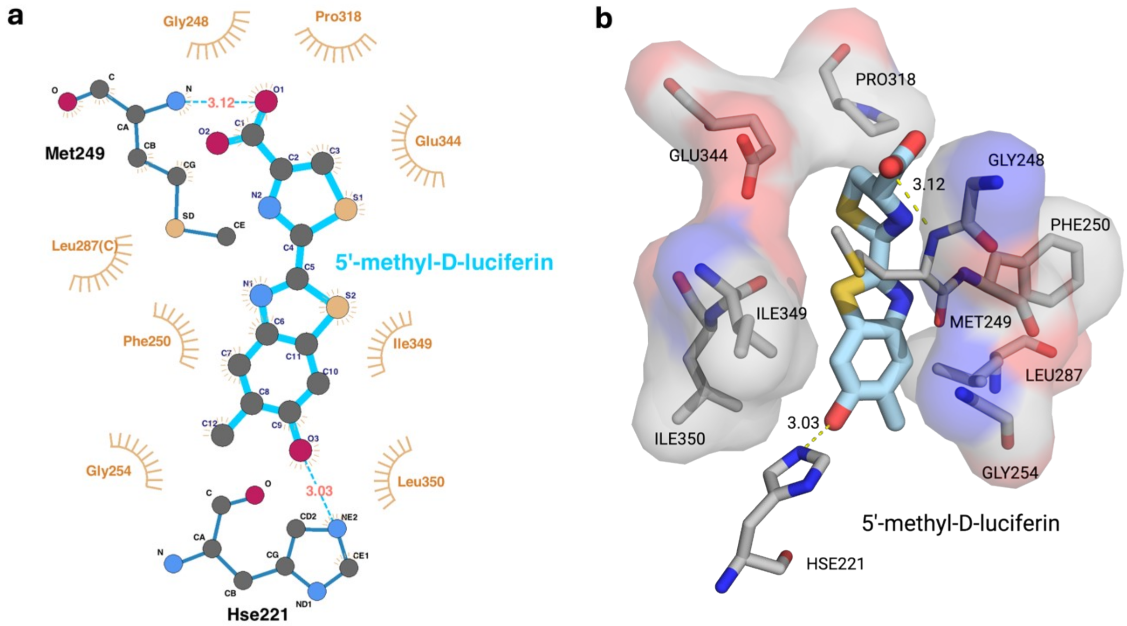

Supplementary Fig. S10. Interactions of active site residues with 5'-methyl-D-luciferin (5'-$MeLH_2$, **2b**). a) The interactions of active site residues with **2b** shown by LigPlot. Hydrogen bonds are shown as blue dashed lines, while the spoked arcs represent protein residues making nonbonded contacts with the ligand. b) 3D structure of active site residues interacting with **2b** based on the LigPlot analysis. The analysis used MD results of temperature at 300K and 40 ns.

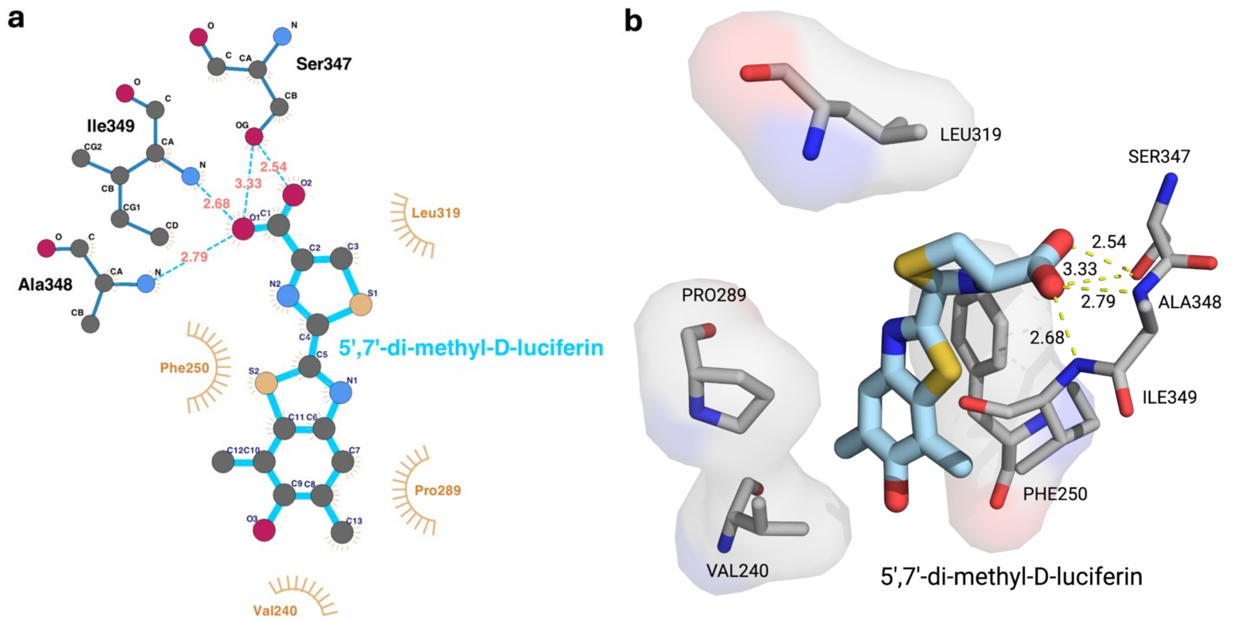

Supplementary Fig. S11. Interactions of active site residues with 5',7'-di-methyl-D-luciferin (5',7'-Di$MeLH_2$, **2c**). a) The interactions of active site residues with **2c** shown by LigPlot. Hydrogen bonds are shown as blue dashed lines, while the spoked arcs represent protein residues making nonbonded contacts with the ligand. b) 3D structure of active site residues interacting with **2c** based on the LigPlot analysis. The analysis used MD results of temperature at 300K and 40 ns.

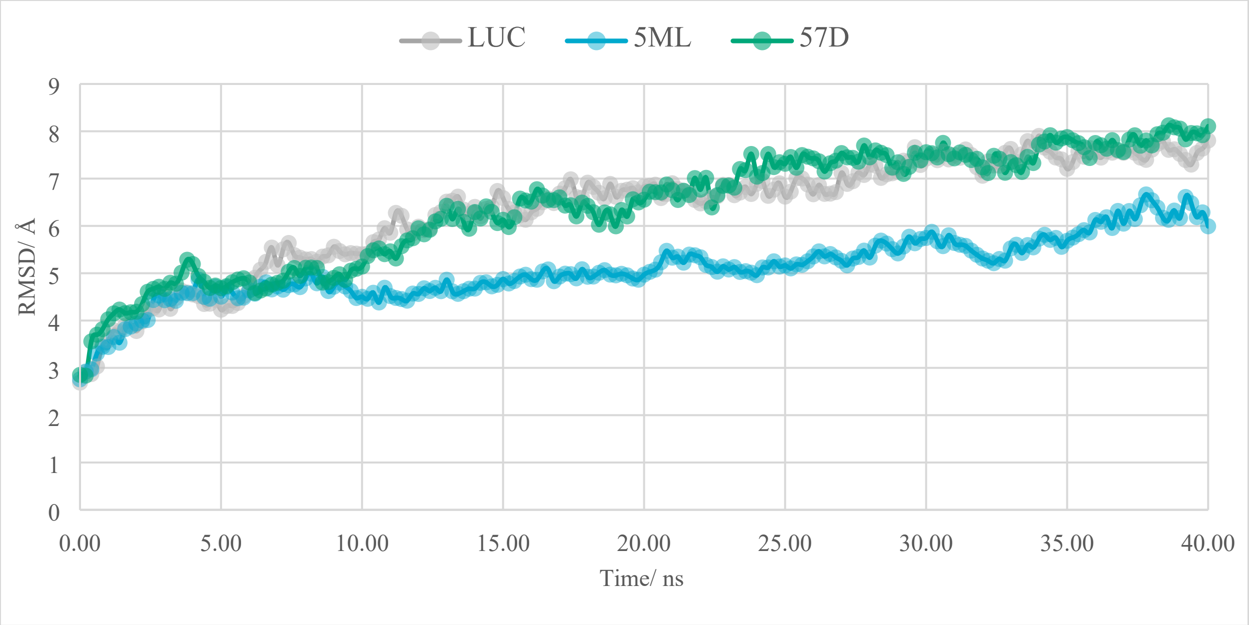

Supplementary Fig. S12. RMSD of Fluc backbone (Carbon (C), α-carbon (CA), and Nitrogen (N) atoms) with various bound luciferins at 300K. LUC, 5ML, and 57D are **2a**, **2b**, and **2c** respectively.

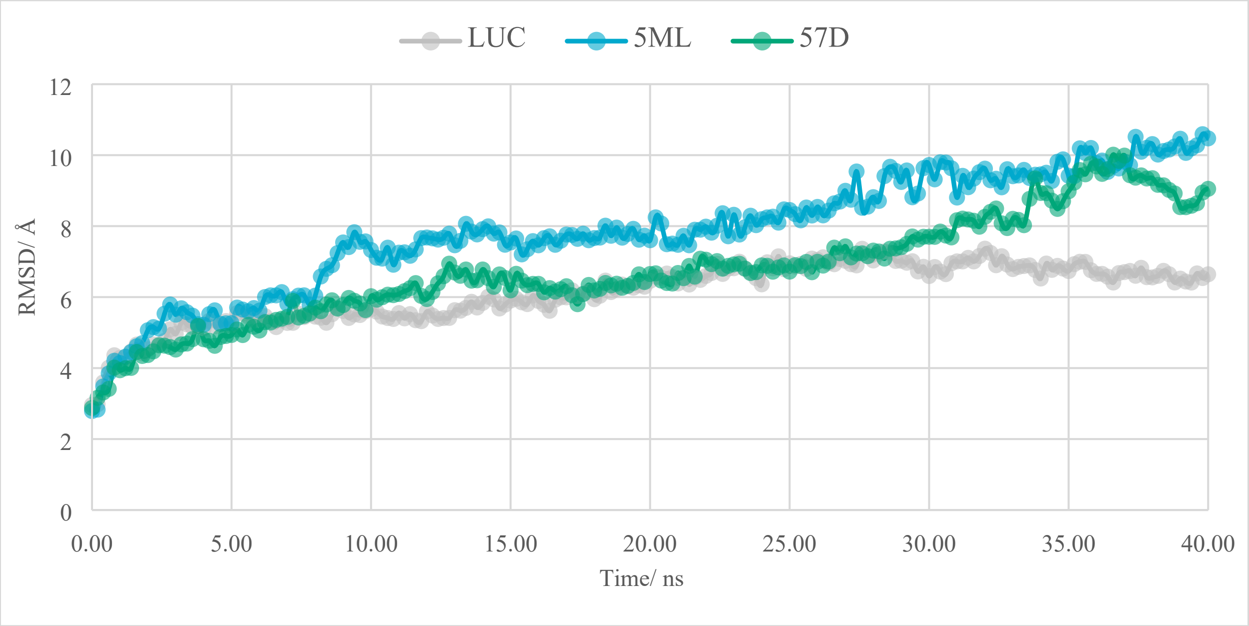

Supplementary Fig. S13. RMSD of Fluc backbone (Carbon (C), α-carbon (CA), and Nitrogen (N) atoms) with various bound luciferins at 320K. LUC, 5ML, and 57D are **2a**, **2b**, and **2c** respectively.

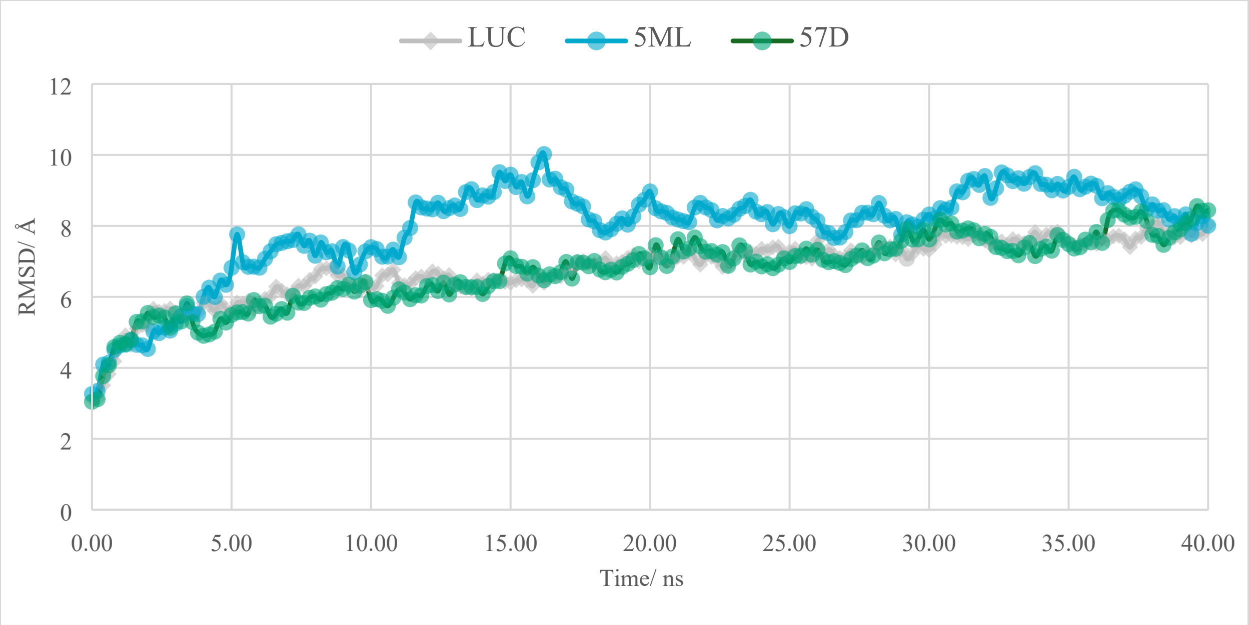

Supplementary Fig. S14. RMSD of Fluc backbone (Carbon (C), α-carbon (CA), and Nitrogen (N) atoms) with various bound luciferins at 340K. LUC, 5ML, and 57D are **2a**, **2b**, and **2c** respectively.

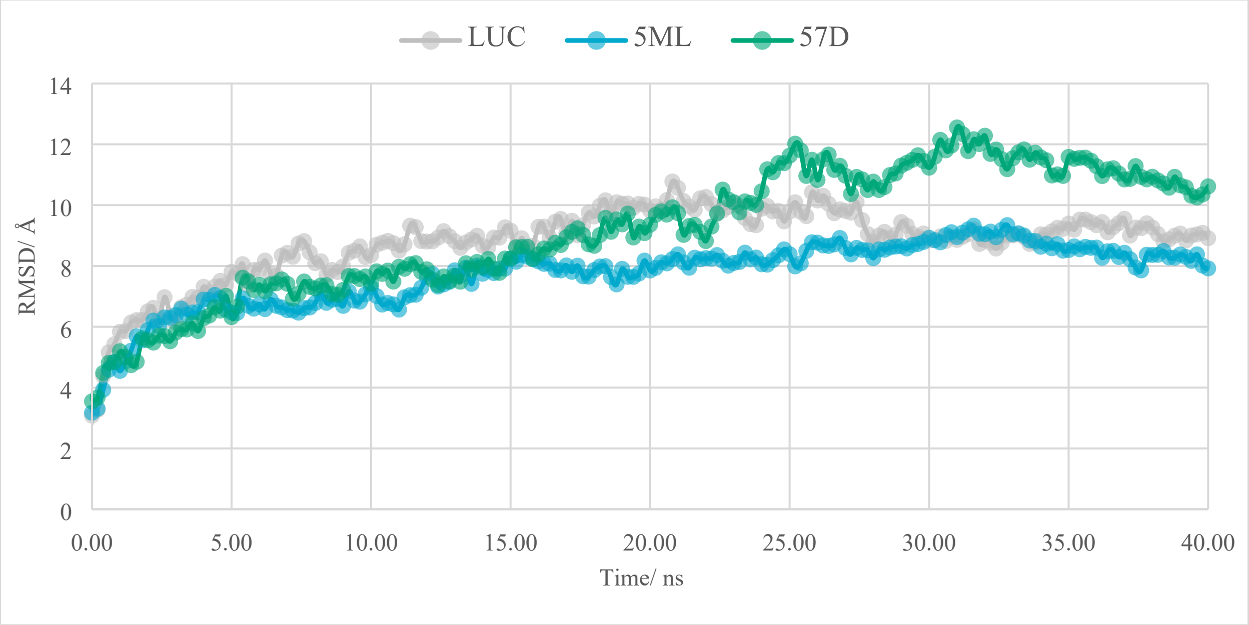

Supplementary Fig. S15. RMSD of Fluc backbone (Carbon (C), α-carbon (CA), and Nitrogen (N) atoms) with various bound luciferins at 360K. LUC, 5ML, and 57D are **2a**, **2b**, and **2c** respectively.

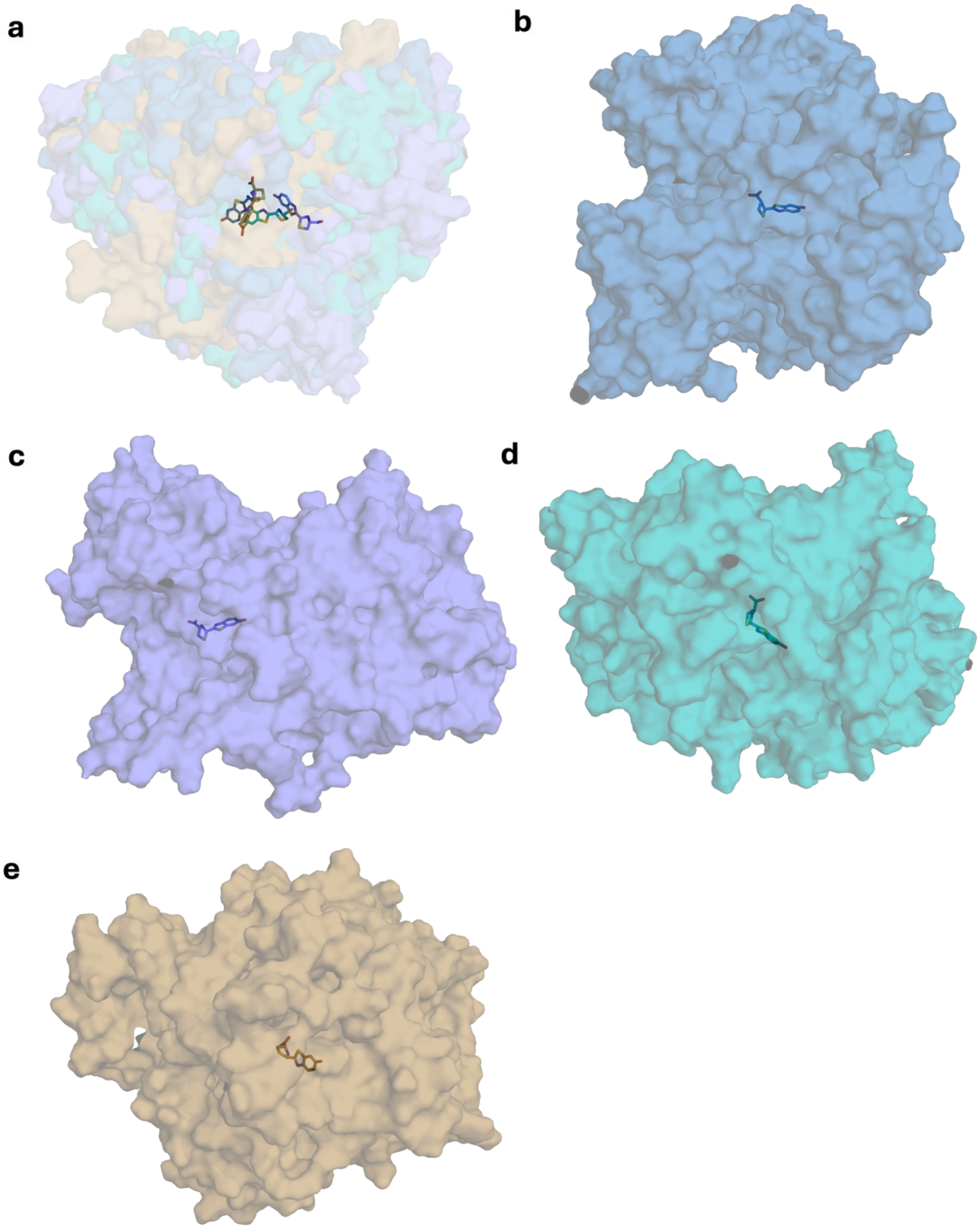

Supplementary Fig. S16. Binding of **2a** in Fluc at various temperatures analyzed by MD simulations. a) Alignment of **2a** binding in Fluc at various temperatures (300-360K) at 40 ns. b) Binding at 300K. c) Binding at 320K. d) Binding at 340K. e) Binding at 360K.

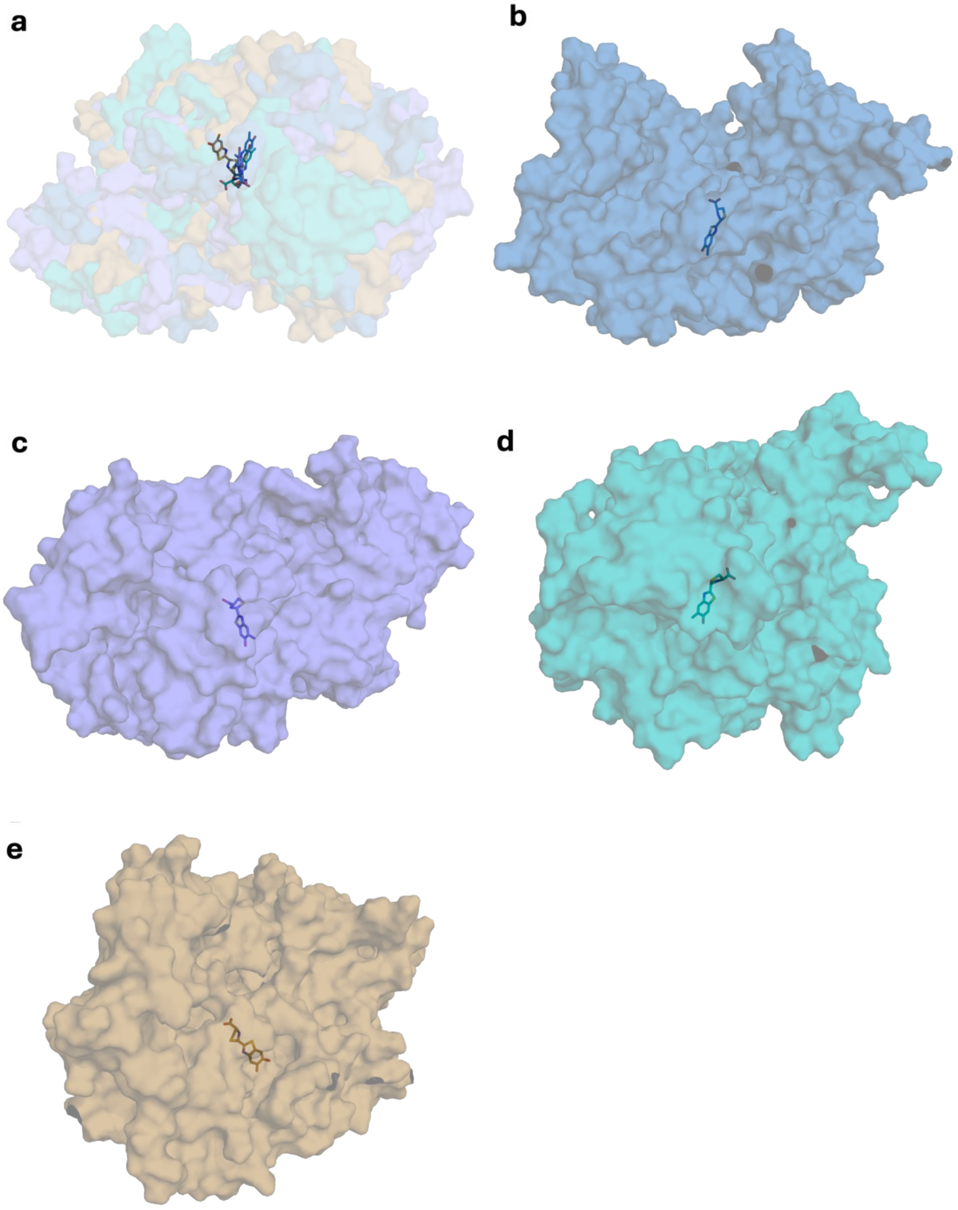

Supplementary Fig. S17. Binding of **2b** in Fluc at various temperatures analyzed by MD simulations. a) Alignment of **2b** binding in Fluc at various temperatures (300-360K) at 40 ns. b) Binding at 300K. c) Binding at 320K. d) Binding at 340K. e) Binding at 360K.

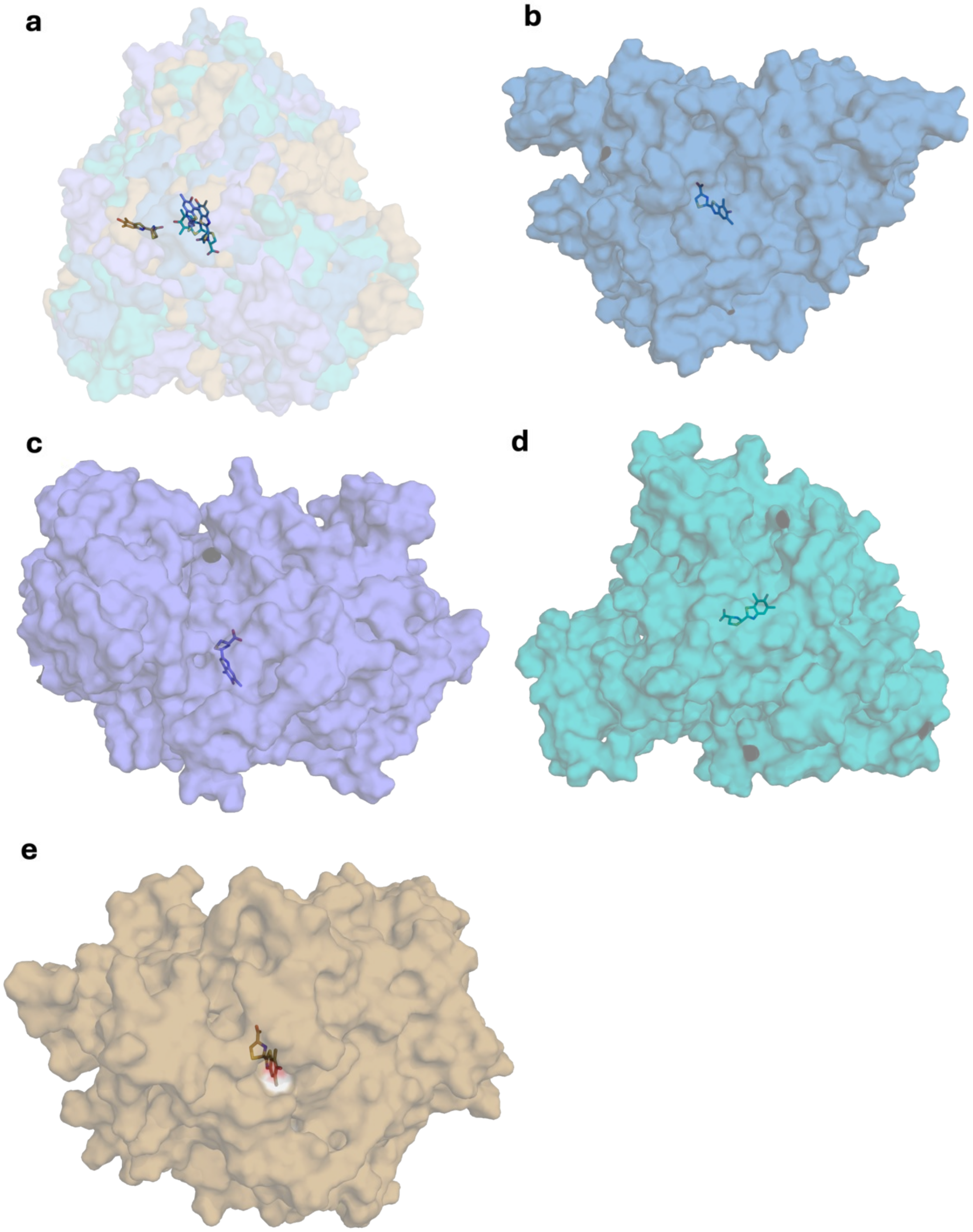

Supplementary Fig. S18. Binding of **2c** in Fluc at various temperatures analyzed by MD simulations. a) Alignment of **2c** binding in Fluc at various temperatures (300-360K) at 40 ns. b) Binding at 300K. c) Binding at 320K. d) Binding at 340K. e) Binding at 360K.

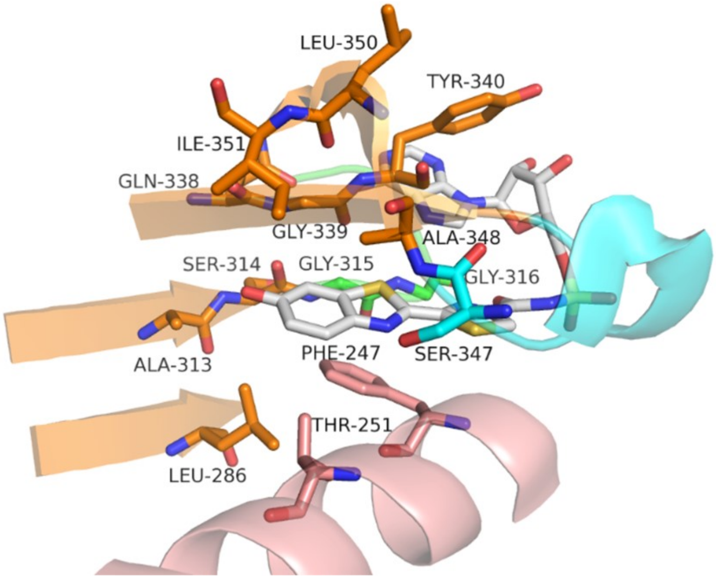

Supplementary Fig. S19. Active site residues of FLuc interacting with 5′-O-[N-dehydroluciferyl)-sulfamoyl]-adenosine (DLSA). Residues within 6 Å of 7'of the bound ATP analogue (DLSA) are shown in gray color. The crystal structure of the firefly luciferase (Fluc) from *Photinus pyralis* with DLSA bound in the active site (PDB: 4G36).

2.12 Comparison of cell line usage in research

We analyzed usage of HepG2, HEK293T, and HeLa cell lines in scientific publications and patents to identify key cell lines used in biomedical research. The key words "HepG2" and "HEK293T" and "HeLa" were used to search Google Scholar to identify publications and patents that used these cells in experiments (the search was performed on December 7, 2024). The use of HeLa and HepG2 cells were significantly higher than that of HEK293T cells. Therefore, we chose to explore applications of D-LH_2 analogues (**2b** and **2c**) in HepG2 and HeLa cells.

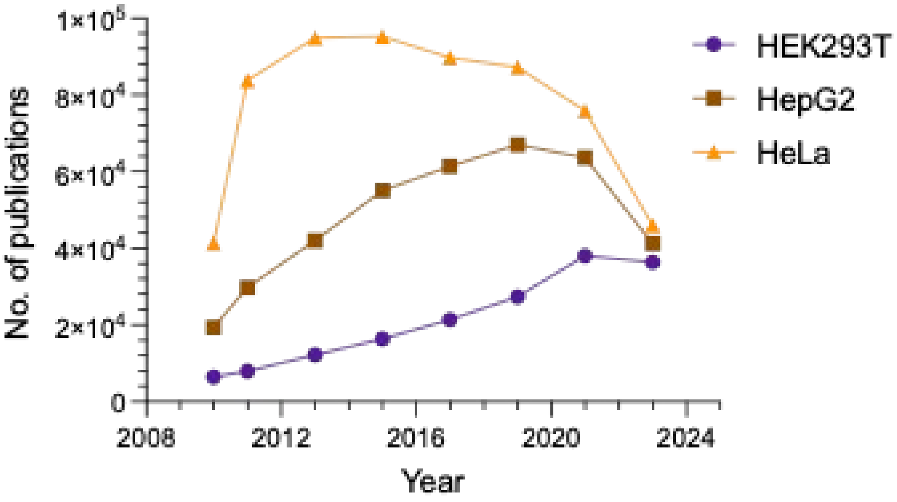

Supplementary Fig. S20 Research articles and patents employing HepG2, HEK293T and HeLa cell lines in experiments.

2.13 Bioluminescence spectra of live HepG2 cells

Bioluminescence spectra of intact Fluc-expressing HepG2 cells mixed with D-LH_2 analogues (**2a**-**2c**) were recorded by a highly sensitive CCD spectrophotometer. Results in Fig. S21 showed that at the same concentration of D-LH_2 analogues, the **2b** compound gave higher BL intensity than those of **2a** and **2c** compounds which showed similar intensity. The raw bioluminescence data were smoothed out using the smooth algorithm in the GraphPad Prism program. The smoothed bioluminescence spectra are shown in Fig. 7a (main text).

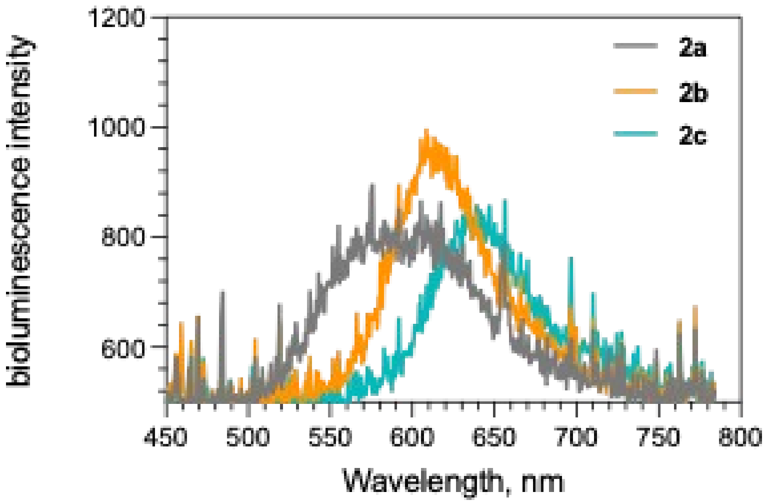

Supplementary Fig. S21. Raw bioluminescence spectra of intact Fluc-expressing HepG2 cells mixed with D-LH_2 analogues (**2a**-**2c**).

2.14 Sensitivity of reporter cells detection

HepG2 cells with stably expressing Fluc and HepG2 cells transiently transfected with plasmids expressing SLR and Pmluc-N230S under the control of the CAG promoter were used as reporter cells. The results in Fig. 7 (main text) and Fig. S22 indicate that the sensitivity of Fluc-expressing HepG2 cells with **2b** gave better sensitivity of detection than those of other compounds when using the F0 filter (no filter). When using the F2 filter (red filter, >620 nm long-pass filter), the **2c** compound provided greater sensitivity than the other compounds with limit of detection (LOD) of cell number about 393 cells. Using HepG2 cells expressing SLR and both F0 and F2 filters, the results in Fig. S22 revealed that the **2c** compound gave greater sensitivity of detection than those of **2a** and **2b** compounds. In these cells, **2b** showed better sensitivity with lower LOD values than those of the natural compound, **2a** in both SLR- and Pmluc-N230S expressing HepG2 cells. Because the **2c** compound provided the low BL signals in the Pmluc-N230S expressing HepG2 cells, the LOD of detection could not be determined (data not shown).

The Fluc, SLR, and Pmluc-N230S plasmids were transiently transfected into HeLa cells to prepare the HeLa reporter cells. Results in Fig. S23 showed that the red-shifted D-LH_2 analogues such as **2b** and **2c** provided greater sensitivity of detection than that of the natural compound (**2a**). Particularly, the **2c** compound gave the greater sensitivity in Fluc-expressing HeLa cells than **2b** and **2a** about 4.1 and 13.0-fold when using F0 filter, respectively. The SLR-expressing HeLa cells showed high sensitivity of detection with **2b** and **2c** compounds than the **2a** compound, especially when using F2 filter. For the Pmluc-N230S expressing HeLa reporter cells, the **2b** compound gave a lower LOD than that of **2a** and its sensitivity is higher than that of **2a** about 2.3 and 1.4-fold when using F0 and F2 filters, respectively (Fig. S23). However, the **2c** compound gave low bioluminescence signals and its LOD in these cells could not be determined (data not shown). All results demonstrated that red-shifted D-LH_2 analogues especially **2b** and **2c** which are promising new substrates of luciferases can provide good bioluminescence signals in living reporter cells with adequate sensitivity of detection with low cell numbers.

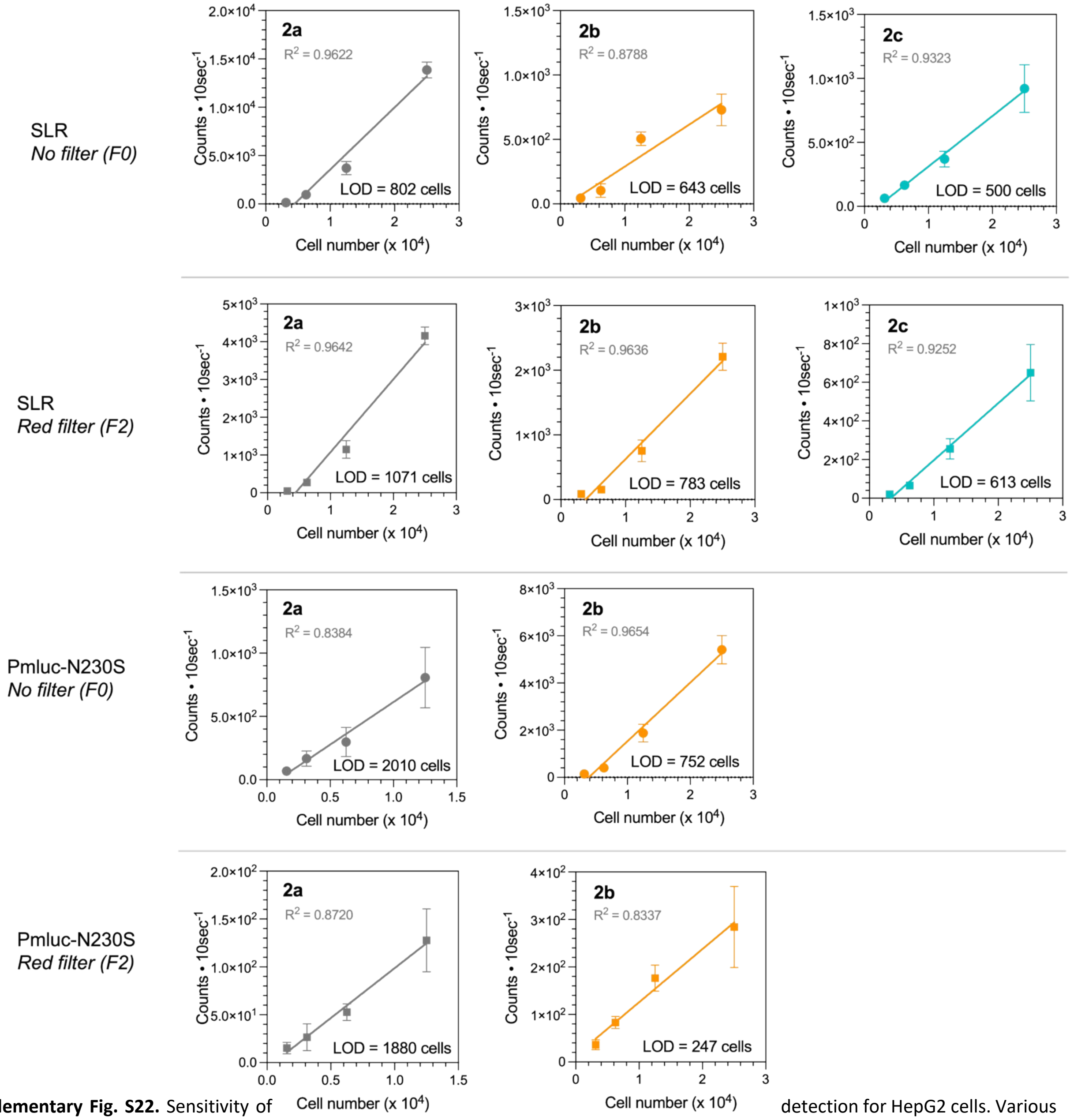

Supplementary Fig. S22. Sensitivity of detection for HepG2 cells. Various cell numbers of HepG2 cells expressing SLR and Pmluc-N230S were placed in each well of microplates. Then, the **2a**, **2b**, **2c** compounds were added into the cells and their bioluminescence signals were measured without destroying cells using F0 (no filter) and F2 (red filter, >620 nm long-pass filter) filters.

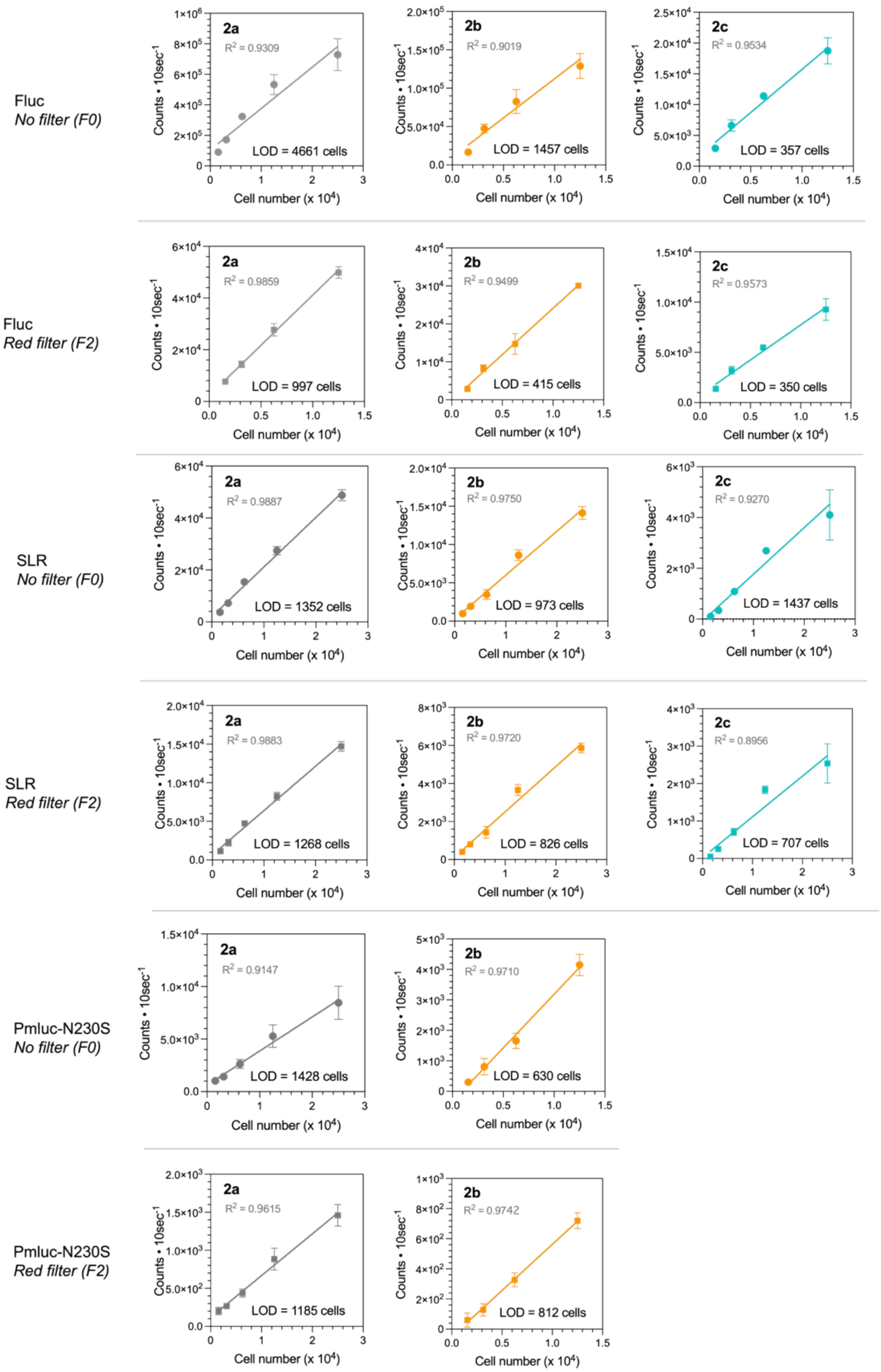

Supplementary Fig. S23. Sensitivity of detection for HeLa cells. Various cell numbers of HeLa cells expressing Fluc, SLR and Pmluc-N230S were placed in each well of microplates. Then, the **2a**, **2b**, **2c** compounds were added into the reporter cells and their bioluminescence signals were measured without destroying cells using F0 (no filter) and F2 (red filter, >620 nm long-pass filter) filters.

2.15 Real-time monitoring of bioluminescence of HeLa cells

HeLa cells expressing Fluc, SLR, and Pmluc-N230S were prepared by transient transfection techniques as described in Section 1.6 and their bioluminescence was monitored in real-time. Results of Fluc-expressing HeLa cells in Fig. S24a-c showed that the **2a** compound (a native D-LH_2) showed the increase of bioluminescence signals up to around 5 hours and the signals gradually decreased after that while for **2b** and **2c,** their bioluminescence signals prolonged much better, up to 13 hours before decreasing (Fig. S24b-c). The SLR-expressing HeLa cells showed similar bioluminescence trend with the Fluc-expressing cells (Fig. S23d-f). The SLR-expressing HeLa cells treated with **2c** showed more prolonged bioluminescence signals (up to 18 hours) than those of **2a** and **2b** compounds (up to 10 hours) before signal decreasing (Fig. S24d-f). For Pmluc-N230S-expressing HeLa cells, all compounds gave much lower bioluminescence signals than in other reporter cells (Fig. S24g-h) with signals of **2c** too low for detection.

When only the red BL signals (F2/F0 ratio) was considered, the results demonstrated that red-shifted D-LH_2 analogues, **2b** and **2c** compounds gave greater red BL signals (highest F2/F0 ratio) than that of native substrate (**2a**), especially for the signals of **2c** in Fluc and SLR-expressing reporter HeLa cells. Therefore, the **2b** and **2c** compounds are potentially new substrates for beetle luciferases, particularly Fluc and SLR for real-time monitoring of cell-based bioluminescence detection with high red BL signals (F2/F0 ratio) and prolonged BL signals in the future.

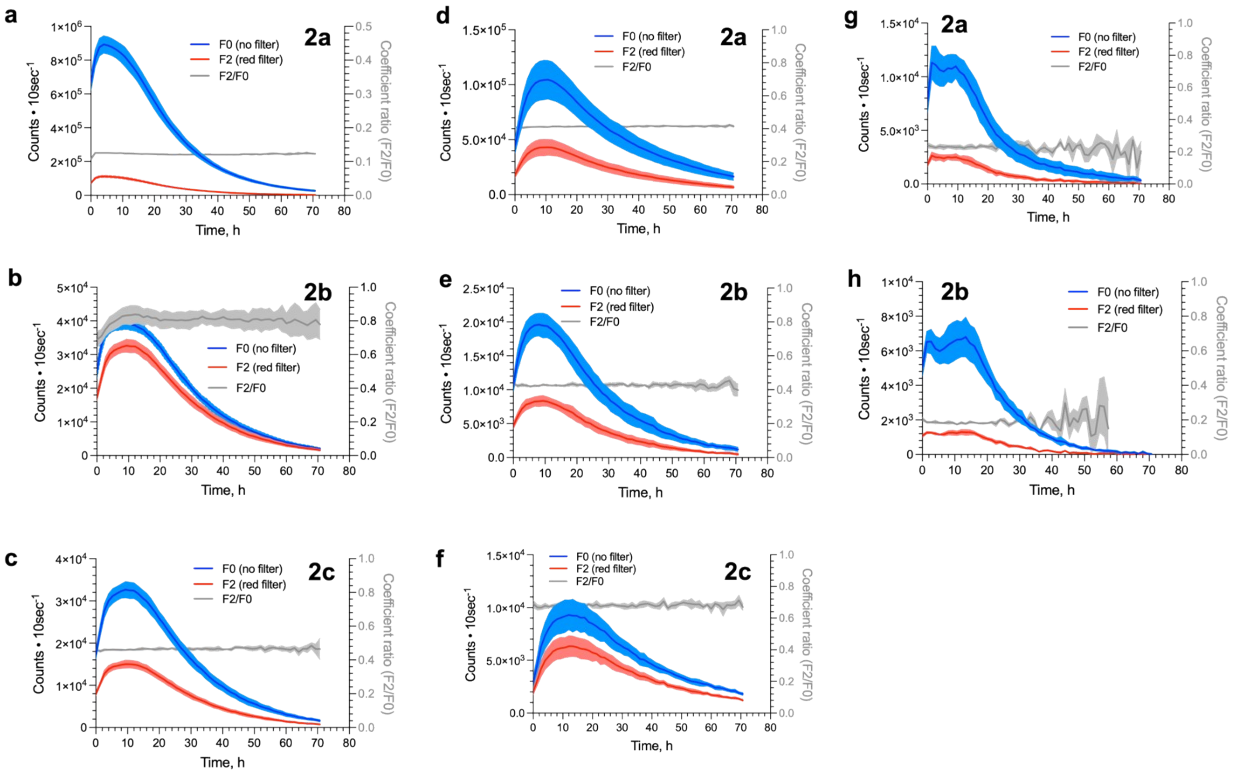

Supplementary Fig. S24. The real-time monitoring bioluminescence of HeLa cells expressing Fluc, SLR, and Pmluc-N230S with **2a**-**2c** compounds. a-c) The real-time monitoring bioluminescence of Fluc-expressing HeLa cells with **2a**, **2b**, and **2c** compounds. d-f) The real-time monitoring bioluminescence of SLR-expressing HeLa cells with **2a**, **2b**, and **2c** compounds. g-h) The real-time monitoring bioluminescence of Pmluc-N230S-expressing HeLa cells with **2a**, and **2b** compounds.

References

1. M. Kato, K. Tsuchihashi, S. Kanie, Y. Oba and T. Nishikawa, *Scientific Reports*, 2024, **14**, 30461.
2. K. Niwa, Y. Ichino, S. Kumata, Y. Nakajima, Y. Hiraishi, D.-i. Kato, V. R. Viviani and Y. Ohmiya, *Photochemistry and Photobiology*, 2010, **86**, 1046-1049.
3. K. Niwa, Y. Ichino and Y. Ohmiya, *Chemistry Letters*, 2010, **39**, 291-293.
4. K. Uno, K. Murotomi, Y. Kazuki, M. Oshimura and Y. Nakajima, *Luminescence*, 2018, **33**, 616-624.
5. S. Yamaguchi, Y. Kazuki, Y. Nakayama, E. Nanba, M. Oshimura and T. Ohbayashi, *PLOS ONE*, 2011, **6**, e17267.
6. Y. Tabei, K. Murotomi, A. Umeno, M. Horie, Y. Tsujino, B. Masutani, Y. Yoshida and Y. Nakajima, *Food and Chemical Toxicology*, 2017, **107**, 129-137.
7. M. Takiguchi, Y. Kazuki, K. Hiramatsu, S. Abe, Y. Iida, S. Takehara, T. Nishida, T. Ohbayashi, T. Wakayama and M. Oshimura, *ACS Synthetic Biology*, 2014, **3**, 903-914.
8. S. Wakuri, K. Yamakage, Y. Kazuki, K. Kazuki, M. Oshimura, S. Aburatani, M. Yasunaga and Y. Nakajima, *Analytical Biochemistry*, 2017, **522**, 18-29.
9. J. A. Sundlov, D. M. Fontaine, T. L. Southworth, B. R. Branchini and A. M. Gulick, *Biochemistry*, 2012, **51**, 6493-6495.
10. G. M. Morris, R. Huey and A. J. Olson, *Current Protocols in Bioinformatics*, 2008, **24**, 8.14.11-18.14.40.
11. M. F. Sanner, *Journal of molecular graphics & modelling*, 1999, **17 1**, 57-61.
12. T. J. Dolinsky, J. E. Nielsen, J. A. McCammon and N. A. Baker, *Nucleic Acids Research*, 2004, **32**, W665-W667.
13. O. Trott and A. J. Olson, *Journal of Computational Chemistry*, 2010, **31**, 455-461.
14. R. B. Best, X. Zhu, J. Shim, P. E. M. Lopes, J. Mittal, M. Feig and A. D. MacKerell, Jr., *Journal of Chemical Theory and Computation*, 2012, **8**, 3257-3273.
15. G. G. Dodson, D. P. Lane and C. S. Verma, *EMBO reports*, 2008, **9**, 144-150-150.
16. E. V. Bocharov, A. G. Sobol, K. V. Pavlov, D. M. Korzhnev, V. A. Jaravine, A. T. Gudkov and A. S. Arseniev, *Journal of Biological Chemistry*, 2004, **279**, 17697-17706.
17. R. Day and V. Daggett, *Journal of Molecular Biology*, 2007, **366**, 677-686.
18. K. Prakinee, A. Phintha, S. Visitsatthawong, N. Lawan, J. Sucharitakul, C. Kantiwiriyawanitch, J. Damborsky, P. Chitnumsub, K.-H. van Pée and P. Chaiyen, *Nature Catalysis*, 2022, **5**, 534-544.
19. J. C. Phillips, R. Braun, W. Wang, J. Gumbart, E. Tajkhorshid, E. Villa, C. Chipot, R. D. Skeel, L. Kalé and K. Schulten, *Journal of Computational Chemistry*, 2005, **26**, 1781-1802.
20. P. Pongpamorn, P. Watthaisong, P. Pimviriyakul, A. Jaruwat, N. Lawan, P. Chitnumsub and P. Chaiyen, *ChemBioChem*, 2019, **20**, 3020-3031.
21. M. Avalos, R. Babiano, N. Cabello, P. Cintas, M. B. Hursthouse, J. L. Jiménez, M. E. Light and J. C. Palacios, *The Journal of Organic Chemistry*, 2003, **68**, 7193-7203.
22. S. Kanie, T. Nishikawa, M. Ojika and Y. Oba, *Scientific Reports*, 2016, **6**, 24794.
23. D. C. McCutcheon, W. B. Porterfield and J. A. Prescher, *Organic & Biomolecular Chemistry*, 2015, **13**, 2117-2121.
24. K. A. Jones, W. B. Porterfield, C. M. Rathbun, D. C. McCutcheon, M. A. Paley and J. A. Prescher, *Journal of the American Chemical Society*, 2017, **139**, 2351-2358.
25. E. H. White, E. Rapaport, H. H. Seliger and T. A. Hopkins, *Bioorganic Chemistry*, 1971, **1**, 92-122.
26. S. J. Williams, C. S. Hwang and J. A. Prescher, *Biochemistry*, 2021, **60**, 563-572.
